# The effects of exercise training on body composition in postmenopausal women: a systematic review and meta-analysis

**DOI:** 10.3389/fendo.2023.1183765

**Published:** 2023-06-14

**Authors:** Mousa Khalafi, Aref Habibi Maleki, Mohammad Hossein Sakhaei, Sara K. Rosenkranz, Mohammad Javad Pourvaghar, Mahsa Ehsanifar, Hadis Bayat, Mallikarjuna Korivi, Yubo Liu

**Affiliations:** ^1^ Department of Physical Education and Sport Sciences, Faculty of Humanities, University of Kashan, Kashan, Iran; ^2^ Department of Exercise Physiology and Corrective Exercises, Faculty of Sport Sciences, Urmia University, Urmia, Iran; ^3^ Department of Exercise Physiology, Faculty of Sport Sciences, University of Guilan, Rasht, Guilan, Iran; ^4^ Department of Kinesiology and Nutrition Sciences, University of Nevada Las Vegas, Las Vegas, NV, United States; ^5^ Department of Exercise Physiology, Central Tehran Branch, Islamic Azad University, Tehran, Iran; ^6^ Institute of Human Movement and Sports Engineering, Zhejiang Normal University, Jinhua, Zhejiang, China

**Keywords:** exercise training, body composition, fat mass, muscle mass, menopause

## Abstract

**Introduction:**

We conducted a systematic review and meta-analysis to investigate the effect of exercise training on body composition outcomes in postmenopausal women.

**Methods:**

PubMed, Web of Science, CINAHL, and Medline were searched to identify the randomized controlled trials which evaluated effect of exercise training versus control in postmenopausal women. Standardized mean differences (SMD), weighted mean differences (WMD) and 95% confidence intervals (95% CIs) were calculated using random effects model.

**Results:**

One hundred and one studies involving 5,697 postmenopausal women were included in the meta-analysis. Results indicated that exercise training effectively increased muscle mass/ volume, muscle and fiber cross-sectional area and fat-free mass, and decreased fat mass, body fat percentage, waist circumference and visceral fat. Furthermore, subgroup analyses results revealed that aerobic and combined training had greater beneficial effects on fat mass outcomes, whereas resistance and combined training had greater beneficial effects on muscle mass outcomes.

**Discussion:**

Overall, our results revealed that exercise training is effective for improving body composition in postmenopausal women. To be specific, aerobic training is effective on fat loss, whereas resistance training is effective on muscle gain. However, combination of aerobic and resistance trainings may be considered a viable strategy to improve body composition in postmenopausal women.

**Systematic review registration:**

https://www.crd.york.ac.uk/prospero/, identifier CRD42021283425.

## Introduction

The postmenopausal phase in women is a critical stage of aging represented by unavoidable changes in the production of endogenous sex hormones, and results hormonal imbalance (1–3). These hormonal changes are associated with increased risks for developing obesity, metabolic syndrome, type 2 diabetes mellitus, and cardiovascular diseases (CVD) (4–6). During the postmenopausal stage, women may experience a series of physiological changes in several cardiometabolic health outcomes (7, 8). Some of the common changes include increased body weight and fat mass, especially redistribution of body fat toward abdominal areas, which contributes to the development of negative cardiometabolic outcomes (9–11). In this regard, menopausal age in women may be associated with increased prevalence of obesity and obesity-related disorders, including metabolic syndrome (12). In the United States, the prevalence of obesity is approximately 65% among women aged 40 to 65 years (13).

Insufficient physical activity is associated with poor menopausal outcomes and increased health risk during the postmenopausal stage of life (14), while lifestyle interventions with either type of exercise is appropriate and effective in promoting the physiological or psychological outcomes in postmenopausal women (15). As a non-pharmacological strategy, exercise training has been shown to be effective, safe, and important to attenuate the age-induced health adversities, and may attribute to improve cardiometabolic outcomes (16, 17). The beneficial effects of exercise intervention are mainly relied on the type of exercise. Resistance training (RT) is known for improving the muscle strength and mass, as well as benefitting the sarcopenia-related phenotypes (18–21). Aerobic training (AT) is known for improving pulmonary function and decreasing fat mass, especially visceral fat in older adults (22–24). However, it is claimed that AT also improves muscle function and lead to skeletal muscle hypertrophy. Therefore, AT also considered as a viable training method to combat sarcopenia in the elderly population (25–27). Besides, previous meta-analyses have confirmed the beneficial effects of RT on muscle mass (28, 29) and AT on fat mass (22) in older adults.

Although several meta-analyses have explored the effects of exercise training in older adults, yet no meta-analysis focused on postmenopausal women and their physical fitness status. Given that this population is affected by hormonal imbalance during aging, such hormonal changes are associated with poor outcomes in health and fitness related variables. The aim of this systematic review and meta-analysis was to elucidate the effects of exercise training on body composition, including muscle mass, fat-free mass (FFM), fat mass, body fat percentage, waist circumference, and visceral fat in postmenopausal women. Subgroup analyses were conducted for the variables, including age of participants, and duration and type of exercise training (aerobic, resistance, and combined) to identify the influential variable and to emphasize the practical and clinical importance of exercise.

## Methods

This systematic review and meta-analysis was conducted in accordance with the latest guidelines of Preferred Reporting Items for Systematic Review and Meta-Analysis (PRISMA) (30), and the Cochrane Handbook of Systematic Reviews of Interventions (31). This study was registered with PROSPERO International prospective register of systematic reviews (ID: CRD42021283425).

### Systematic search strategy

A systematic search was conducted in electronic databases, including PubMed, Web of Science, CINAHL, and Medline for research published from inception to October 2021 to identify original articles using the following search strategy: (“menopausal” or “post menopause” or “post-menopause” or “menopause” or “elderly women” or “older women”) AND (“exercise” or “exercise training” or “physical activity”). The search strategy was adapted for each database and was conducted using “AND” and “OR” Boolean operators. When available in the respective databases, limitations were applied for English language, human participants, article/document type, and randomized controlled trials. In addition, reference lists of all retrieved records and previous meta-analyses (32, 33) were screened for relevant articles. After removing duplicate publications, the titles, abstracts, and keywords of the remaining studies were screened to assess the study eligibility for full-text review against inclusion and exclusion criteria. Then, the full-texts of the studies that met criteria were further screened. The search strategy and screening processes were conducted independently by two authors (AM and MS), and any disagreements were resolved through discussion with another author (MKh).

### Inclusion and exclusion criteria

According to the PRISMA latest guidelines (30) and our study purpose, we have followed these criteria to include or exclude the articles. Inclusion criteria were as follows: (a) English language, peer-reviewed articles; (b) randomized controlled trials that included exercise training versus non-exercise (control) groups; (c) studies on postmenopausal women; (d) studies measured the main outcomes at baseline and post-intervention; and (e) intervention durations ≥ 4 weeks. In order to maximize generalizability, participants included middle-aged to older women who were postmenopausal, ranging from healthy (absence of disease diagnosis) to frail with chronic diseases. Exercise training modalities included any mode of exercise training, such as aerobic training, resistance training, combined training, functional training, yoga, high-intensity interval training (HIIT) and Tai chi. For the main outcomes, studies were included that measured at least one of the following body composition item: muscle mass and volume, muscle and fiber cross-sectional area (CSA), fat-free mass (FFM) (or lean mass if FFM was not available), fat mass, body fat percentage, waist circumference, and visceral fat. Body composition outcomes were measured by magnetic resonance imaging (MRI), computerized tomography (CT), ultrasound, densitometry, dual energy X-ray absorptiometry (DXA), hydro-densitometry, or In-body and/or whole-body air plethysmography (BodPod) (34). Waist circumference was measured by tape and recorded in cm or inches. Exclusion criteria include non-English, non-full text articles (conference abstracts), intervention with a duration of less than 4 weeks, and non-original studies.

### Data extraction and synthesis

Two reviewers (A H M and M H S) independently extracted the following data from each included study: 1) study characteristics, including study design and year of publication; 2) participant characteristics, including sample size, biological sex, health status, age, and body mass index (BMI); 3) intervention characteristic, including training type, intensity, frequency, duration; and supervision of exercise sessions; 4) outcome variables and assessment methodologies; 5) pre- and post-intervention means and standard deviations (SD), or mean changes and their SD values for outcomes. When required, the means and SDs were calculated from the reported standard errors, medians, ranges and/or interquartile ranges as described previously (31, 35, 36). In addition, when required, Getdata Graph Digitizer software was used for extracting data from figures (37). For studies with multiple intervention arms, all comparisons were included and subsequently the sample size of the repeated intervention was divided by the number of comparisons to avoid double counting. Furthermore, for studies that did not provide sufficient information, we have contacted the corresponding author of the relevant articles.

### Quality assessment and sensitivity analysis

The methodological quality for each included study was assessed by two independent reviewers (AM and MS) using the Physiotherapy Evidence Database (PEDro) tool (38), and any disagreements were resolved through discussion with another author (MKh). This tool examined the following domains: eligibility criteria, random allocation of participants, allocation concealment, group similarity at baseline, blinding of participants, blinding of intervention providers, assessors blinded, outcome measures assessed in 85% of participants, intention-to-treat analysis, reporting of between groups statistical comparisons and point measures, and measures of variability reported for main effects. However, we excluded 2 items including blinding of participants and intervention providers because these could not feasibly be blinded with regard to assigned exercise conditions during studies, and this may not influence the quality of studies (39). Therefore, study quality was assessed based on the remaining 9 items. Each source of bias was judged as low, high, or unclear (due to insufficient detail) (**Supplementary Table 2**). In addition, sensitivity analyses were performed by omitting each study individually to determine whether results changed significantly.

### Statistical analysis

Meta-analysis was conducted using random effects models using the DerSimonian and Laird approach (39) to calculate standardized mean differences (SMD) or weighted mean differences (WMD) and 95% confidence intervals (CIs) for comparing the effects of exercise training versus control on muscle mass and volume, muscle and fiber CSA, FFM, fat mass, body fat percentage, visceral fat mass, and waist circumference. In addition, several sub-group analyses were performed based on age (middle-aged: <65yrs and older adults: ≥65yrs), type of training (aerobic, resistance, combined) and intervention duration (medium-term: ≤16 weeks, long-term: >16 weeks). Subgroup analyses were performed when there were more than 3 interventions for each subgroup. Interpretation of effect sizes was conducted using Cochrane guidelines as follows: 0.20–0.49 indicating small effect size, 0.5–0.79 indicating medium effect size, and >0.8 indicating large effect size (40). Statistical heterogeneity was evaluated using Cochran Q tests and I^2^ statistics as follows: 25% indicating low heterogeneity, 50% indicating moderate heterogeneity, and 70% indicating high heterogeneity (41). Publication bias was assessed with visual interpretation of funnel plots and Egger’s tests as secondary determinants of bias at a cut-point of p<0.10 (42). In addition, trim and fill correction was used to address the potential effects of publication bias where relevant (43).

## Results

### Included study characteristics

**Figure 1:**
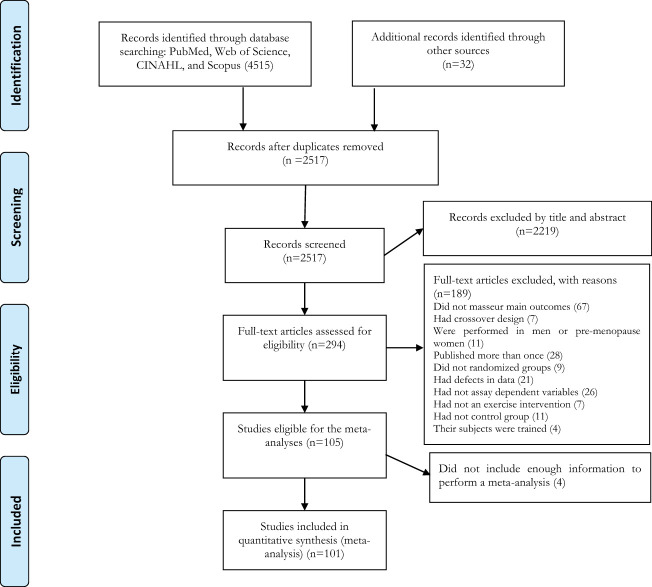
Flow diagram of systematic literature search.

The search strategy retrieved 990 records from PubMed, 1,290 records from Web of Science, 942 records from CINAHL, and 1,292 records from MEDLINE. After examination for duplicates, 1,998 articles were excluded, and then 2,223 articles were excluded after reviewing the titles and abstracts. A total of 294 articles were identified for full-text assessment based on inclusion and exclusion criteria. An additional 196 articles were excluded due to the reasons presented in **Figure 1**. Finally, 101 articles of randomized controlled trials with parallel arm-trials were included in the meta-analysis (**Figure 1**).

### Participant characteristics

A total of 5,697 postmenopausal women were included in the meta-analysis. The mean age of participants was ranged from 51 to ~89 yrs., and the mean BMI was ranged from 21 to 34 kg.m^2^. Sample size of individual studies was ranged from 14 to 320 participants. To increase the generalizability of our meta-analysis results, postmenopausal women regardless of their health status, comprised a wide range of health (absence of disease) and chronic disease characteristics (metabolic diseases, cardiovascular diseases, cancer, and osteoporosis) were included. Full details of participant characteristics are summarized in **Supplementary Table 1**.

### Intervention characteristics

Exercise training characteristics are summarized in **Supplementary Table 1**. All included studies compared the effects of exercise training versus a control group using random allocation. Intervention durations of included studies was ranged from 4 weeks to 18 months, while frequency of exercise sessions was ranged from 1 to 7 per week, with three sessions being the most common. For type of exercise training, most of the included studies conducted aerobic, resistance, or combined training, and others used water-based exercise, yoga, Tai chi, Pilates, yoga and Korean dance, and functional training. Exercise training was supervised in several studies, while other studies followed both supervised and unsupervised exercise training during the intervention period. However, supervision details were not clearly reported in few studies.

### Meta-analysis

#### Body composition

##### Muscle mass

**Figure 2:**
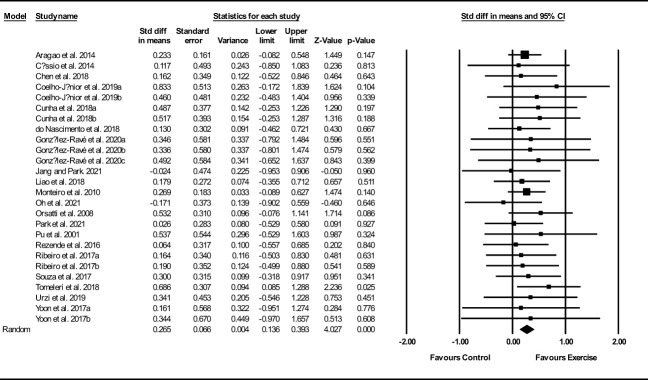
Forest plot of the effects of exercise training versus control on muscle mass. Data are reported as SMD (95% confidence limits). SMD, standardized mean difference.

Based on 26 intervention arms, exercise training increased muscle mass/volume (SMD: 0.26; 95% CI: 0.13, 0.39; P=0.001) (**Figure 2**). There was no significant heterogeneity amongst the included studies (I^2 = ^0.00%; p=0.99). Visual interpretation of funnel plots suggested publication bias, but the Egger’s test did not indicate bias was present (p=0.35). After accounting missing studies (5 studies) with the trim and fill method, the overall change was 0.20 (95% CI: 0.08, 0.32). In addition, sensitivity analysis by omitting individual studies showed that significance did not change. Subgroup analyses revealed a significant increase in muscle mass in middle-aged (SMD: 0.26, p=0.01) and older adults (SMD: 0.26, p=0.001), with resistance training (SMD: 0.27, p=0.001), combined training (SMD: 0.26, p=0.02), in medium-term interventions (SMD: 0.26, p=0.002) and long-term interventions (SMD: 0.26, p=0.008) (**Supplementary Table 3**).

##### Muscle and fiber CSA

**Figure 3:**
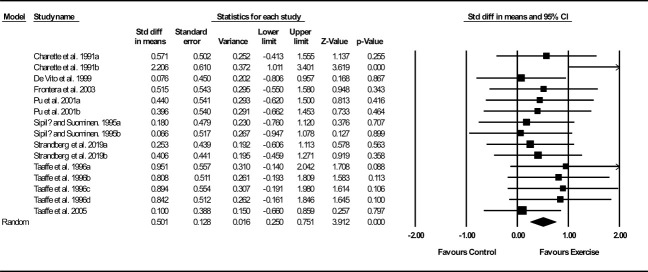
Forest plot of the effects of exercise training versus control on muscle and fiber CSA. Data are reported as SMD (95% confidence limits). SMD, standardized mean difference.

Based on 15 intervention arms, exercise training increased muscle and fiber CSA (SMD: 0.50; 95% CI: 0.25, 0.75; P=0.001) (**Figure 3**). There was no significant heterogeneity amongst included studies (I^2 = ^0.00%; p=0.49). Visual interpretation of funnel plots did not suggest publication bias, but the Egger’s test did indicate that bias was likely (p=0.002). In addition, sensitivity analysis by omitting individual studies showed that significance did not change. Subgroup analyses revealed a significant increase in muscle mass in older adults (SMD: 0.59, p=0.001), with resistance training (SMD: 0.57, p=0.001), in medium-term interventions (SMD: 0.64, p=0.02) and long-term interventions (SMD: 0.44, p=0.005) (**Supplementary Table 3**).

##### FFM

**Figure 4:**
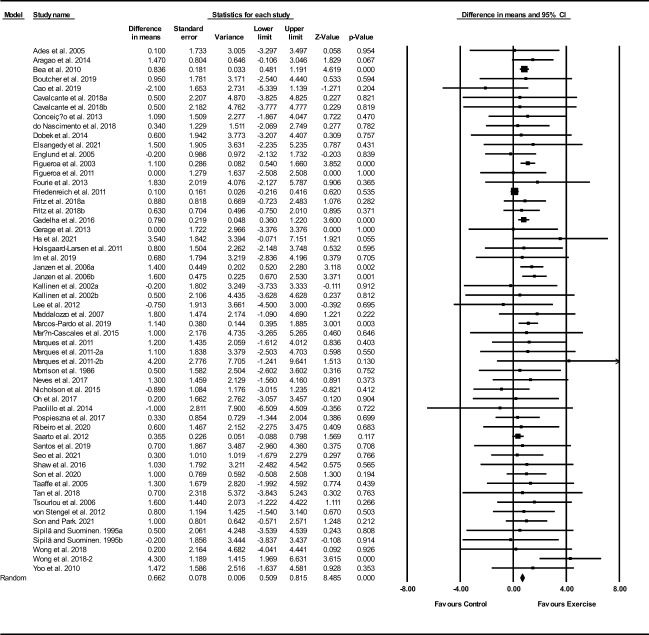
Forest plot of the effects of exercise training versus control on FFM. Data are reported as WMD (95% confidence limits). WMD, weighted mean difference.

Based on 56 intervention arms, exercise training increased FFM (WMD: 0.66 kg; 95% CI: 0.50, 0.81; P=0.001) (**Figure 4**). There was no significant heterogeneity amongst included studies (I^2 = ^0.00%; p=0.62). Visual interpretation of funnel plots suggested publication bias, but the Egger’s test did not indicate bias was present (p=0.18). After accounting missing studies (6 studies) with the trim and fill method, the overall change was 0.66 kg (95% CI: 0.45, 0.87). In addition, sensitivity analysis by omitting individual studies showed that significance did not change. Subgroup analyses revealed a significant increase in FFM mass in middle-aged (WMD: 0.71 kg, p=0.001) and older adults (WMD: 0.86 kg, p=0.001), with resistance training (WMD: 0.90 kg, p=0.001), combined training (WMD: 0.68 kg, p=0.001), water-based training (WMD: 2.49 kg, p=0.005), in medium-term interventions (WMD: 0.83 kg, p=0.001) and long-term interventions (WMD: 0.79 kg, p=0.001) (**Supplementary Table 3**).

##### Fat mass

**Figure 5:**
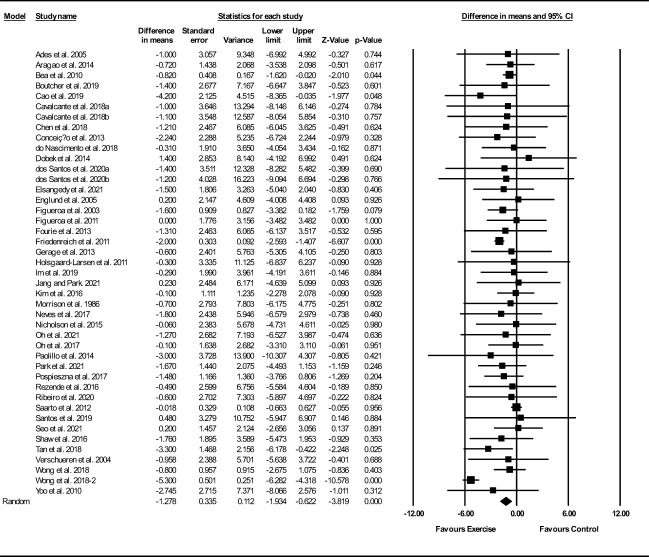
Forest plot of the effects of exercise training versus control on fat mass. Data are reported as WMD (95% confidence limits). WMD, weighted mean difference.

Based on 43 intervention arms, exercise training decreased fat mass (WMD: -1.27 kg; 95% CI: -1.93, -0.62; P=0.001) (**Figure 5**). There was significant heterogeneity amongst included studies (I^2 = ^56.46%; p=0.001). Visual interpretation of funnel plots suggested publication bias, but the Egger’s test did not indicate bias was present (p=0.54). After accounting missing studies (16 studies) with the trim and fill method, the overall change was -2.63 kg (95% CI: -2.63, -1.38). Sensitivity analysis by omitting individual studies showed that significance did not change. Subgroup analyses revealed a significant decrease in fat mass in middle-aged adults (WMD: -1.15, p=0.001), with aerobic training (WMD: -1.94, p=0.001), in medium-term interventions (WMD: -1.17, p=0.002), and long-term interventions (WMD: -1.24, p=0.02) (**Supplementary Table 3**).

##### Body fat percentage

**Figure 6:**
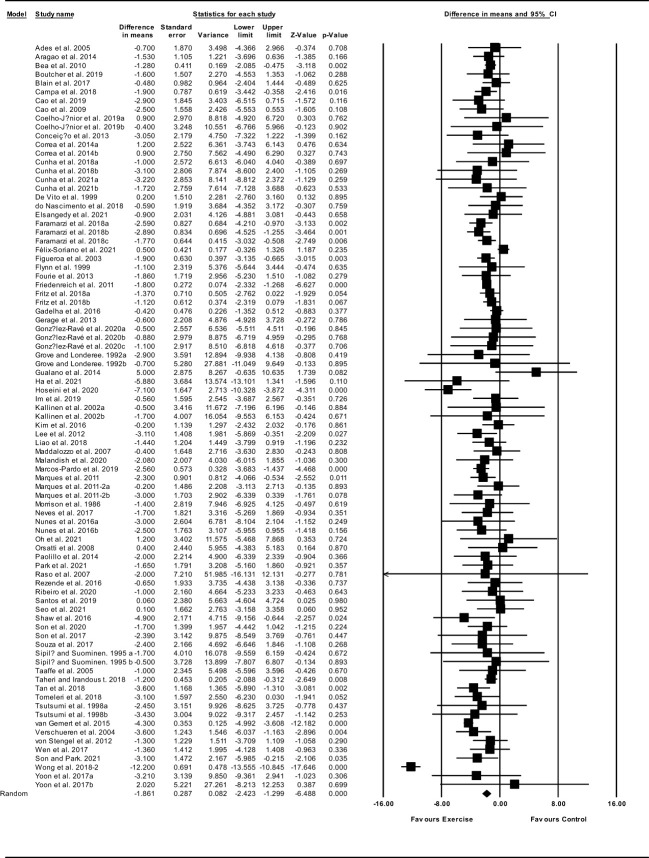
Forest plot of the effects of exercise training versus control on body fat percentage. Data are reported as WMD (95% confidence limits). WMD, weighted mean difference.

Based on 85 intervention arms, exercise training decreased body fat percentage (WMD: -1.86%; 95% CI: -2.42, -1.29; P=0.001) (**Figure 6**). There was significant heterogeneity amongst included studies (I^2 = ^77.20%; p=0.001). Visual interpretation of funnel plots suggested publication bias, but the Egger’s test did not indicate bias was present (p=0.59). After accounting missing studies (28 studies) with the trim and fill method, the overall change was -2.59% (95% CI: -3.11, -2.06). In addition, sensitivity analysis by omitting individual studies showed that significance did not change. Subgroup analyses revealed a significant decrease in fat percentage in middle-aged adults (WMD: -1.92%, p=0.001) and older adults (WMD: -1.76%, p=0.001), with resistance training (WMD: -1.20%, p=0.001), aerobic training (WMD: -1.68%, p=0.001), combined training (WMD: -2.24%, p=0.001), in medium-term interventions (WMD: -1.79%, p=0.001) and long-term interventions (WMD: -1.82%, p=0.001) (**Supplementary Table 3**).

##### Waist circumference

**Figure 7:**
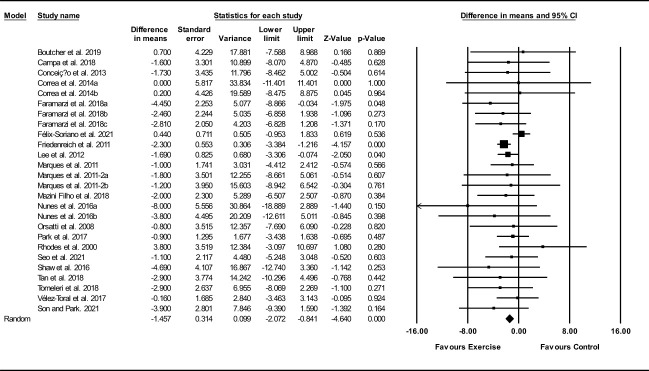
Forest plot of the effects of exercise training versus control on waist circumference. Data are reported as WMD (95% confidence limits). WMD, weighted mean difference.

Based on 26 intervention arms, exercise training decreased waist circumference (WMD: -1.45 cm; 95% CI: -2.05, -0.83; P=0.001) (**Figure 7**). There was no significant heterogeneity amongst included studies (I^2 = ^0.00%; p=0.79). Visual interpretation of funnel plots suggested publication bias, but the Egger’s test did not indicated that bias was present (p=0.63). After accounting missing studies (3 studies) with the trim and fill method, the overall change was -1.35 cm (95% CI: -1.96, -0.74). In addition, sensitivity analysis by omitting individual studies showed that significance did not change. Subgroup analyses revealed a significant decrease in waist circumference in middle-aged (WMD: -1.42 cm, p=0.001), older adults (WMD: -1.50 cm, p=0.04), with aerobic training (WMD: -2.30 cm, p=0.001), combined training (WMD: -1.66 cm, p=0.03), in medium-term interventions (WMD: -2.69 cm, p=0.001) and long-term interventions (WMD: -1.18 cm, p=0.002) (**Supplementary Table 3**).

##### Visceral fat

**Figure 8:**
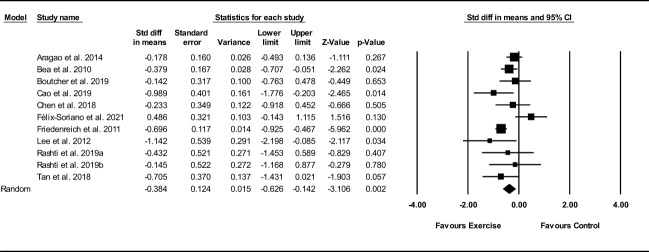
Forest plot of the effects of exercise training versus control on visceral fat. Data are reported as SMD (95% confidence limits). SMD, standardized mean difference.

Based on 11 intervention arms, exercise training decreased visceral fat (SMD: -0.38; 95% CI: -0.62, -0.14; P=0.002) (**Figure 8**). There was significant heterogeneity amongst included studies (I^2 = ^53.64%; p=0.01). Visual interpretation of funnel plots suggested publication bias, but the Egger’s test did not indicate bias was present (p=0.61). After accounting missing studies (1 studies) with the trim and fill method, the overall change was -0.34 (95% CI: -0.59, -0.10). In addition, sensitivity analysis by omitting individual studies showed that significance did not change.

## Discussion

In this meta-analysis with a large sample size, we have assessed the effects of exercise training on body composition, including muscle mass, muscle and fiber CSA, lean mass or fat-free mass, fat mass, body fat percentage, waist circumference, and visceral fat in postmenopausal women. Our main findings revealed that exercise training positively influenced the body composition components, including muscle mass, muscle fiber CSA, FFM, fat mass, body fat percentage, waist circumference, and visceral fat in postmenopausal women. Greater beneficial effects on fat mass outcomes were evidenced with aerobic training, whereas greater beneficial effects on muscle mass outcomes were reported with resistance training. In addition, a majority of these beneficial effects appears to be occurred with medium- and long-term interventions and also in middle-aged and older postmenopausal women.

### Muscle mass outcomes

The loss of muscle mass is considered to be an important contributor of strength loss in older adults with advancing age (44). Menopausal period is associated with loss of muscle mass and muscle strength, which may progress to sarcopenia over a period of time (45), and this phenomenon is primarily linked with natural decrease of estrogen in postmenopausal women (46–48). Natural decline in estrogen was reported to cause endocrine dysfunction, metabolic syndrome, decreased bone mass density, muscle mass and strength, and increased visceral fat mass (45, 48). Nevertheless, loss of muscle mass due to age cannot be ruled-out as older men also represented with higher prevalence of sarcopenia. Previous studies have shown sex-specific absolute loss of muscle loss, where elderly men are likely to have more muscle mass than elderly women, but tend to lose muscle mass faster (49–51). Although men experienced greater loss of absolute muscle mass, women experienced greater decrements in muscle quality (52). In this context, either type of exercise training is a practical strategy to prevent or delay the age-induced loss of muscle mass in men and women.

Previous reviews and meta-analyses have determined the effectiveness of exercise training and indicated that exercise is a one of the best approach to prevent and treat the muscle weakness in older adults (53–56), however less is known about such benefits among postmenopausal women specifically. Of particular importance for postmenopausal women with a high risk for sarcopenia, our results confirmed the positive effects of exercise training on muscle mass. Although aerobic training may also have minimal effects on muscle size (57), our results suggested that resistance training is important for increasing muscle mass, and did not indicate significant increases for aerobic training interventions. Nevertheless, combined training was similarly effective as compared with resistance training. These results are consistent with previous meta-analyses indicating that resistance training increased muscle mass in older adults and even very old adults (28, 56). Although older men may gain more absolute muscle size in response to resistance exercise training, there are no biological sex differences in relative muscle strength gains (54). The similar adaptations may be due to the fact that neither protein synthesis nor mTOR signaling differ between the biological sexes following resistance training (54). Our results indicate that combined training is also effective for increasing muscle mass and FFM, suggesting that in postmenopausal women, muscle mass development can also be improved by combining resistance training with aerobic training. In addition, our results suggested that muscle mass and FFM were increased irrespective of age groups in postmenopausal women. These adaptions are consistent with previous reviews suggesting the positive effects of resistance training in middle-aged, older, and very old adults (28, 56, 58). In addition, subgroup analysis based on intervention duration (medium-term: <16 weeks and long-term: ≥16 weeks), increased muscle mass and FFM occurred regardless of intervention duration. This results shows that exercise training with duration <16 weeks can be also important for improving muscle. However, it should be noted that muscle fiber CSA results should be interpreted with caution due to the small number of studies in some subgroups.

### Fat mass outcomes

Despite the fact that exercise training is effective in reducing the fat mass, evidence regarding the types of exercise training in postmenopausal women is scarce. Although exercise training combined with diet has been shown to be an effective strategy for weight loss and fat mass reduction, regardless of exercise type, some systematic reviews and meta-analyses concluded that exercise interventions effectively reduced fat mass (59–63). In general, our results suggested that exercise training is effective for reducing the adiposity markers including fat mass, body fat percentage, visceral fat, and waist circumference. The potential mechanism for reductions in adiposity are related to altered energy balance where energy is expended during exercise as well as shortly after exercise as the body recovers, and increases in resting metabolic rate that follow increased lean body mass (64). However, it is important to note that the type of exercise is important as a moderator of the effectiveness of exercise training on fat mass. In this regard, previous systematic reviews have shown that aerobic training is more effective in reducing body weight, fat mass, and waist circumference when compared to resistance training in individuals with BMIs ≥ 25 kg/m^2^ (65). In line with a systematic review conducted by Schwingshackl and colleagues (65), our results confirmed that aerobic training was effective in reducing fat mass, with small effects for resistance training (-0.45 kg) and not reaching statistical significance (p=0.06). Reductions in fat mass and related indicators following aerobic training interventions may be due to energy expenditure during the exercise bouts, which is likely to be higher as compared with resistance training (65, 66). In addition, we found that both aerobic and resistance trainings are effective in reducing body fat percentage. However, it should be noted that body fat percentage, particularly following resistance training interventions may include reduced fat mass as well as increased FFM, and our results also showed a significant increase in FFM with resistance training. Furthermore, we found that aerobic training is effective in reducing waist circumference and visceral fat, which was not the case for resistance training with regard to waist circumference. Visceral fat is known to be an important risk factor for many chronic diseases such as type 2 diabetes and cardiovascular diseases (60). In addition, waist circumference is considered as a surrogate clinical measure for visceral (abdominal) fat mass (67). In our study, there were a small number of studies that determined visceral fat, and therefore we could not perform subgroup analysis. But subgroup analysis based on exercise type, revealed significant reductions in waist circumference (-2.30 cm) occurred with aerobic training. The results for aerobic training were obtained from 3 studies, whereas there were 16 studies included for resistance exercise, which should be considered when interpreting the results. Furthermore, our results indicated that combined training is effective for decreasing body fat percentage and waist circumference, suggesting that this type of training may be a suitable strategy for optimization of the combination of both fat loss and muscle gain in postmenopausal women. For better understanding the role of participants’ age and intervention duration on exercise-induced fat loss, subgroup analyses were conducted, and found that fat loss adaptations following training occurred regardless of age and intervention duration. These results are important, especially regarding age factor, indicating the effectiveness of exercise training for postmenopausal women at any age. Exercise training is also considered to be effective intervention for improving musculoskeletal health by a positive effect on bone mineral density (68, 69). Given that the increase in fat mass and the loss LBM affects on bone mineral density in postmenopausal women (70), exercise training may have a positive effect on bone mineral density by improving body composition.

### Limitations

Our study has limitations that should be considered when interpreting the results. For outcome assessments, included studies measured body composition using different methods, which may lead to differences in reported results. There were significant heterogeneities among included studies with respect to some outcomes that may be due to differences in exercise interventions, participant characteristics, and the quality of the included studies. We did not include any limitations regarding the health of participants, and non-communicable chronic diseases such as obesity and type 2 diabetes may influence exercise training adaptations. In addition, we did not include any limitations on the age of participants. However, we performed subgroup analysis on middle-aged and older adults, showing positive effects of exercise regardless of age. Finally, we did not include bone mineral density as a outcomes

## Conclusions

The current systematic review and meta-analysis demonstrated that exercise training is effective in improving the body composition in postmenopausal women, represented by increased muscle mass and decreased fat mass, regardless of age and intervention duration. In addition, our results confirmed that aerobic exercise is more beneficial on fat loss, while resistance exercise is more beneficial on muscle gain. Since body composition includes both lean and fat tissue, a combination of aerobic and resistance exercise may be beneficial to promote overall health among older women. Additional studies on the effectiveness of combined training in postmenopausal women depends on their physical fitness may be necessary before recommendations.

## Data availability statement

The original contributions presented in the study are included in the article/**Supplementary Material**. Further inquiries can be directed to the corresponding authors.

## Author contributions

MKh, AM, MS, MP, SR, MKo and YL conceived and designed the study. MKh, AM, MS, HB and ME extracted the data. MKh, AM, MS, MKo and YL analyzed the data and completed the initial draft of the results. MKh, MKo and YL drafted the initial manuscript. And SR, MP, MKo and YL revised the manuscript. All authors approved the final version of the manuscript.
