# Supplementary material for: The effects of exercise training on body composition in postmenopausal women: a systematic review and meta-analysis
Source: Front Endocrinol (Lausanne). 2023 Jun 14;14:1183765. doi: 10.3389/fendo.2023.1183765 (PMC10306117; doi:10.3389/fendo.2023.1183765)
Supplement: Supplementary file 1 [file Table_1.docx]

**Supplementary Table 1**. Participant and Intervention Characteristics

| Source, year | Participant Characteristics | | | | Intervention Characteristics | | | | | Outcomes  (measurement method) |
| --- | --- | --- | --- | --- | --- | --- | --- | --- | --- | --- |
|  | Sample size | Participant Health Status | Age [years] | BMI [kg/m2] | Exercise Type | Description of Exercise Intervention | Supervised or Unsupervised | Duration | Frequency  (d/week) |  |
| Ades et al., 2005 [1] | 42 | disabled with coronary heart disease | EX:72.9±6.1  CON:71.5±4.8 | EX:28.7±4.2  CON:30.2±5.6 | Resistance | whole body exercises; 1-2 sets with 10 reps at 50-80% of 1RM | Supervised | 6 months | 3 | FM, FP, and FFM (DEXA) |
| Aragão et al., 2014 [2] | 254 | healthy | EX:55.4±5.1  CON:56.9±4.9 | EX:27.9±4.4  CON:28.6±4.9 | Combined | 20-25 min at 50-84% of HRR + whole body exercises; 2-4 sets with 8-12 reps at 70-80% of 1RM | Supervised | 12 months | 3 | FM, FP, FFM, VF, and MM (InBody) |
| Bea et al., 2010 [3] | 148 | healthy | EX:55.8±0.5  CON:56.4±0.6 | EX:25.3±3.8  CON:25.3±3.8 | Resistance | whole body exercises; 2 sets with 6-8 reps at 70-80% of 1RM | Supervised | 12 months | 3 | FM, FP, VF, LBM (DEXA) |
| Blain et al., 2017 [4] | 121 | physically deconditioned | EX:65.6±4.4  CON:65.8±4.2 | EX:24.3±3.5  CON:26.6±4.2 | Aerobic | 50-min at 40-80% of HR_max_ | Supervised & Unsupervised | 6 months | 3 | FP (DEXA) |
| Boutcher et al., 2019 [5] | 40 | overweight | EX:54.1±3.6  CON:53.3±3.4 | EX:28.3±3.7  CON:27.3±4.1 | HIIT | 60 reps of 8-s at near-maximal exertion with 12-s recovery at 80-85% of HR_max_ | Supervised | 8 weeks | 3 | FM, FP, VF, LBM (DEXA), WC |
| Campa et al., 2018 [6] | 30 | healthy | EX:66.5±4.3  CON:65.6±5.2 | EX:28.8±4.6  CON:32.4±5.6 | Resistance | whole body suspension exercises; 4 sets with 12 reps | Supervised | 12 weeks | 2 | FP (caliper), WC |
| Cao et al., 2019 [7] | 30 | overweight and obese | EX:63.8±5.9  CON:64.0±4.6 | EX:28.0±2.9  CON:26.4±1.4 | Aerobic | 20-40 min at the individualized FAT max HR | Supervised | 12 weeks | 3 | FM, FP, FFM (DEXA), VF (InBody) |
| Cao et al., 2009 [8] | 126 | healthy | EX:63.2±4.5  CON:66.5±4.3 | EX:23.7±2.2  CON:22.5±2.3 | Combined | aerobic exercise, antigravity exercise, circuit training, and resistance exercises; lower body resistance exercises; 1-2 sets with 20-30 reps | Supervised & Unsupervised | 12 months | 3-4 | FP (ND) |
| Cavalcante et al., 2018 [9] | 63 | overweight and obese | EX_1_:67.6±5.7  EX_2_:66.5±6.0  CON:66.7±4.1 | EX_1_:29.1±4.5  EX_2_:29.6±4.9  CON:28.2±4.8 | Resistance | whole body exercises; 1 set with 10-15 reps at 10-15 RM | Supervised | 12 weeks | EX_1_: 2  EX_2_: 3 | FM, FFM (DEXA) |
| Charette et al., 1991 [10] | 27 | healthy | EX:69.8±4.3  CON:67.7±4.2 | ND | Resistance | resistance exercises; 3-6 sets with 6 reps at 65-75% of 1RM | Supervised | 12 weeks | 3 | CSA (biopsy) |
| Chen et al., 2018 [11] | 33 | sarcopenia | EX:66.7±5.3  CON:68.3±2.8 | ND | Resistance | whole body kettlebell training; 3 sets with 8-12 reps at 60-70% of 1RM | Supervised | 8 weeks | 2 | FM, MM, VF (InBody) |
| Coelho-Júnior et al., 2019 [12] | 45 | healthy | EX_1_:67.0±6.2  EX_2_:66.7±5.1  CON:66.7±4.6 | EX_1_:30.2±4.1  EX_2_:27.8±6.2  CON:27.0±7.7 | Resistance | EX_1_: non-periodized resistance exercises; 1-3 sets of 8-15 reps at a rating of 5-10 BRPE  EX_2_: daily undulating periodization resistance exercises; 1-3 sets of 8-15 reps at a rating of 5-10 BRPE | Supervised | 22 weeks | 2 | FP, MM (InBody) |
| Conceição et al., 2013 [13] | 20 | healthy | EX:53.4±3.9  CON:53.0±5.7 | EX:26.2±3.3  CON:25.3±1.8 | Resistance | whole body exercises; 3 sets with 8-10 reps | Supervised | 16 weeks | 3 | WC, FM, FP, LBM (caliper) |
| Correa et al., 2014 [14] | 35 | healthy | EX_1_:59.5±6.3  EX_2_:59.5±6.3  CON:59.5±6.3 | EX_1_:25.4±4.1  EX_2_:26.1±3.6  CON:25.7±2.6 | Resistance | EX_1_: whole body exercises; 3 sets at 15RM  EX_2_: whole body exercises; 1 set at 15RM | Supervised | 12 weeks | 5 | WC, FP (caliper) |
| Cunha et al., 2018 [15] | 68 | osteo-sarcopenic obesity | EX_1_:66.6±5.1  EX_2_:68.3±4.2  CON:67.3±3.6 | EX_1_:27.1±4.3  EX_2_:26.7±4.8  CON:26.7±4.6 | Resistance | EX_1_: whole body single-set exercises; 1 set with 10-15 reps  EX_2_: whole body multiple-sets exercises; 3 sets with 10-15 reps | Supervised | 12 weeks | 3 | FP, MM (DEXA) |
| Cunha et al., 2021 [16] | 65 | healthy | EX_1_:70.3±6.3  EX_2_:68.7±4.7  CON:69.0±4.2 | EX_1_:27.9±5.2  EX_2_:26.5±5.0  CON:26.5±4.5 | Resistance | EX_1_: whole body single-set exercises; 1 set with 10-15 reps  EX_2_: whole body multiple-sets exercises; 3 sets with 10-15 reps | Supervised | 12 weeks | 3 | FP (DEXA) |
| De Vito et al., 1999 [17] | 22 | healthy | EX:63.0±3.1  CON:63.5±3.3 | ND | Aerobic | 20-25 min walking linked with upper- and lower-limb exercise at 60% of HRR | Supervised | 12 weeks | 3 | FP (caliper), CSA (ultrasound) |
| do Nascimento et al., 2018 [18] | 44 | healthy | EX:66.3±4.8  CON:66.4±4.0 | EX:24.9±3.3  CON:26.2±2.6 | Resistance | whole body exercises; 2 sets with 10 reps until moderate fatigue in each exercise or when it began to get difficult | Supervised | 12 weeks | 3 | MM, FM, FP, FFM (DEXA) |
| Dobek et al., 2014 [19] | 67 | breast cancer survivors | EX:64.2±6.0  CON:63.8±7.3 | EX:28.9±5.5  CON:27.9±4.5 | Resistance + impact training | whole body dumbbell and barbells and weighted vests exercises; 2 sets with 8-12 reps | Supervised & Unsupervised | 12 months | 3 | FM, LBM (DEXA) |
| dos Santos et al., 2020 [20] | 59 | healthy | EX_1_:67.3±4.4  EX_2_:67.3±4.4  CON:67.3±4.4 | EX_1_:27.6±5.0  EX_2_:27.6±5.0  CON:27.6±5.0 | EX_1_: resistance with narrow repetition-zone  EX_2_: resistance with wider zone | EX_1_: whole body exercises; 3 sets with 12/10/8 reps at 12/10/8 RM-load  EX_2_: whole body exercises; 3 sets with 15/10/5 reps at 15/10/5 RM-load | Supervised | 8 weeks | 3 | FM (DEXA) |
| Duff et al., 2017 [21] | 44 | healthy | EX:65.3±4.6  CON:65.0±4.7 | ND | Resistance | whole body exercises; 2 sets with 8-12 reps to fatigue | Supervised | 9 months | 3 | MM (DEXA) |
| Elsangedy et al., 2021 [22] | 32 | healthy | EX:65.7±3.3  CON:66.3±2.8 | EX:26.5±3.6  CON:25.3±3.3 | Resistance | self-selected resistance exercises: 1-3 sets with 5-10 reps at low to high load | Supervised | 12 weeks | 3 | FFM, FM, FP (DEXA) |
| Englund et al., 2005 [23] | 48 | healthy | EX:72.8±3.6  CON:73.2±4.9 | EX:25.2±2.7  CON:26.1±3.2 | Combined | 10-min steps combinations with coordinated arm movements + lower and core resistance exercises; 2 sets with 8-12 reps + static balance and coordination | Supervised | 12 months | 2 | FM, LBM (DEXA) |
| Faramarzi et al., 2018 [24] | 40 | overweight | EX_1_:60.3±0.8  EX_2_:60.3±0.8  EX_3_:60.3±0.8  CON:60.3±0.8 | EX_1_:29.9±1.2  EX_2_:29.2±1.7  EX_3_:27.6±0.9  CON:31.7±0.9 | EX_1_: aerobic - resistance  EX_2_: resistance – aerobic  EX_3_: alternative combined | A: 16-30 min at 60-88% of HR_max_  R: whole body exercises; 2-3 sets with 8-18 reps at 40-75% of 1RM | Supervised | 10 weeks | 3 | WC, FP (caliper) |
| Félix-Soriano et al., 2021 [25] | 42 | overweight and obese | EX:59.0±3.5  CON:58.8±3.4 | EX:30.8±2.3  CON:30.2±2.3 | Resistance | whole body exercises; 3-4 sets with 8-15 reps at 50-80% of 1RM | Supervised | 16 weeks | 2 | WC, FP, VF (DEXA) |
| Figueroa et al., 2003 [26] | 94 | healthy | EX:57.4±4.4  CON:57.8±4.8 | ND | Combined | A: 25-min at 50-80% of HR_max_  R: whole body exercises; 2 sets with 8 reps at 70-80% of 1RM | Supervised | 12 months | 3 | FM, FP, LBM (DEXA) |
| Figueroa et al., 2011 [27] | 24 | healthy | EX:54.0±6.9  CON: 54.0±3.5 | EX:24.2±2.4  CON:23.1±2.4 | Combined | A: 20-min at 60% of HR_max_  R: whole body circuit exercises; 1 set with 12 reps at 60% of 1RM | Supervised | 12 weeks | 3 | FM, FFM (InBody) |
| Flynn et al., 1999 [28] | 29 | healthy | EX:72.6±3.5  CON:72.9±4.9 | EX:25.6±4.4  CON:26.3±2.0 | Resistance | lower body exercises; 3 sets with 8 reps at 70-80% of 1RM | Supervised | 10 weeks | 3 | FP (ND) |
| Fourie et al., 2013 [29] | 50 | healthy | EX:66.1±4.8  CON:65.3±5.0 | EX:28.3±6.8  CON:29.3±5.4 | Pilates | 60-min Pilates included breathing, followed by a flow from standing, to sitting, to lying down exercises | Supervised | 8 weeks | 3 | FM, FP, LBM (caliper) |
| Friedenreich et al., 2011 [30] | 320 | healthy | EX:61.2±5.4  CON:60.6±5.7 | EX:29.1±4.5  CON:29.2±4.3 | Aerobic | 15-45 min at 70-80% of HRR | Supervised & Unsupervised | 12 months | 5 | WC, FM, FP (DEXA), VF, LBM (CT) |
| Fritz et al., 2018 [31] | 75 | overweight | EX_1_:69.2±1.1  EX_2_:70.4±1.0  CON:67.2±1.1 | EX_1_:27.4±1.0  EX_2_:29.0±0.9  CON:26.5±0.8 | Resistance | EX_1_: whole body traditional elastic band exercises; 3-4 sets with 10 reps at a rating of 7-9 OMNI scale  EX_2_: whole body elastic tubes with handles exercises; 3-4 sets with 10 reps at a rating of 7-9 OMNI scale | Supervised | 8 weeks | 2 | FP, FFM (InBody) |
| Frontera et al., 2003 [32] | 14 | healthy | EX:73.7±3.4  CON:74.3±3.8 | EX:23.7±3.6  CON:23.2±1.6 | Resistance | lower body exercises; 4 sets with 8 reps at 65-80% of 1RM | ND | 12 weeks | 3 | CSA (CT) |
| Gadelha et al., 2016 [33] | 133 | healthy | EX:66.8±5.4  CON:67.3±5.0 | EX:27.1±4.0  CON:29.1±5.1 | Resistance | whole body exercises; 3 sets with 8-12 reps at 60-80% of 1RM | Supervised | 24 weeks | 3 | FP, FFM (DEXA) |
| Gerage et al., 2013 [34] | 31 | healthy | EX:65.5±5.0  CON:66.2±4.1 | EX:23.9±2.9  CON:25.1±3.4 | Resistance | whole body exercises; 2 sets with 10-15 reps until moderate fatigue in each exercise or stopped when it began to be diﬃcult | Supervised | 12 weeks | 3 | FFM, FM, FP (DEXA) |
| Cássio et al., 2014 [35] | 17 | hypertensive | EX:65.6±5.0  CON:66.1±3.8 | EX:27.1±4.2  CON:27.8±1.9 | Resistance | whole body exercises; 2 sets with 15 reps at 40% of 1RM | ND | 12 weeks | 3 | MM (DEXA) |
| González-Ravé et al., 2020 [36] | 48 | healthy | EX_1_:65.7±4.5  EX_2_: 65.7±4.5  EX_3_: 65.7±4.5  CON:65.7±4.5 | ND | Combined | EX_1_: 60-70% of HR_max_, resistance exercises; 3 sets with 12-20 reps at 67-70% of 1RM, agility, coordination and flexibility  EX_2_: EX_1_ + 1 session circuit resistance exercises; 2-3 sets with 8-12 reps at 70-80% of 1RM  EX_3_: EX_1_ + 1 session circuit resistance exercises; 2-3 sets with 8-12 reps at a rating of 7-8 BRPE | ND | 10 weeks | EX_1_: 2  EX_2_: 3  EX_3_: 3 | FP, MM (InBody) |
| Grove and Londeree, 1992 [37] | 15 | healthy | EX_1_:54.0±1.9  EX_2_:56.6±4.3  CON:56.0±4.5 | ND | EX_1_: high impact exercise  EX_2_: low impact exercise | Exercises using a force plate  EX_1_: greater than or equal to two times body weight, jumping jacks (3.29 x body weight), running-in-place (2.47 x body weight), and knee-to-elbow with jump (2.79 x body weight)  EX2: less than 1.5 times body weight, slow walk (1.19 x body weight), fast walk (1.49 x body weight), heel jacks without a jump (1.34 x body weight), and the Charleston (1.32 x body weight) | Supervised | 12 months | 3 | FP (Skinfold) |
| Gualano et al., 2014 [38] | 30 | healthy | EX:63.6±3.6  CON:66.3±6.0 | EX:28.2±3.6  CON:26.8±5.5 | Resistance | 3 sets of 8-12 RM, except during the first week of a reduced volume of 2 sets of 15-20 RM | Supervised | 24 weeks | 2 | FP (DEXA) |
| Ha et al., 2021 [39] | 13 | metabolic syndrome | EX:75.8±4.3  CON:76.7±5.9 | EX:23.9±1.5  CON:24.8±1.1 | Water-based exercise | 30-40% heart rate reserve at a rating of RPE 9-10 for weeks 1-5, 40-50% heart rate reserve (RPE 11-12), for weeks 6-10, and 50-60% heart rate reserve (RPE 13-14) for weeks 11-16, 50 min | Supervised | 16 weeks | 3 | LBM, FP (InBody) |
| Holsgaard-Larsen et al., 2011 [40] | 23 | healthy | EX:69.7±3.4  CON:69.7±3.4 | ND | Resistance | Lower body exercises; 4 sets with 8-10 reps at 75–80% of 1RM | Supervised | 12 weeks | 2 | FM, FFM (InBody) |
| Hoseini et al., 2020 [41] | 20 | vitamin D deficiency and NAFLD | EX:62.6±1.9  CON:62.0±1.9 | EX:33.6±1.9  CON:34.7±1.7 | Aerobic | 45-60 min at 60-75% of HR_max_ | Supervised | 8 weeks | 3 | FP (InBody) |
| Im et al., 2019 [42] | 25 | healthy | EX:71.6±3.2  CON:69.4±2.9 | EX:24.8±2.8  CON:26.4±2.4 | Yoga and Korean dance | 60-min yoga and Korean dance at a rating of 12-13 BRPE | Supervised | 12 weeks | 3 | FM, FP, LBM (InBody) |
| Jang and Park, 2021 [43] | 20 | healthy | EX:73.3±4.5  CON:71.9±6.7 | ND | Resistance | Lower body exercises; 1-2 sets with 10 reps | ND | 4 weeks | 3 | FM, MM (InBody) |
| Janzen et al., 2006 [44] | 57 | healthy | EX_1_:54.8±6.5  EX_2_:55.8±8.2  CON:58.8±6.7 | ND | EX_1_: bilateral resistance  EX_2_: unilateral resistance | EX_1_: whole body bilateral resistance exercises; 1-2 sets with 8-12 reps at 50-60% of 1RM  EX_2_: whole body unilateral resistance exercises; 1-2 sets with 12 reps at 50-60% of 1RM | Supervised | 26 weeks | 3 | LBM (DEXA) |
| Kallinen et al., 2002 [45] | 42 | healthy | EX_1_:76-78  EX_2_:76-78  CON:76-78 | ND | EX_1_: aerobic  EX_2_: resistance | EX_1_: 20-40 min at 50-80% of HRR  EX_2_: whole body exercises; 3-4 sets with 8-10 reps at 60-75% of 1RM | Supervised | 18 weeks | 2-3 | FP, LBM (InBody) |
| Kim et al., 2016 [46] | 69 | sarcopenic obesity | EX:81.4±4.3  CON:81.1±5.1 | EX:25.1±2.5  CON:25.3±2.8 | Combined | whole body elastic band, chair and machine exercises; 1-3 sets with 10 reps | Supervised | 3 months | 2 | FM, FP (InBody) |
| Lee et al., 2012 [47] | 16 | obese | EX:54.8±2.8  CON:54.3±2.9 | EX:25.1±1.6  CON:25.2±1.7 | Yoga | 60-min of breathing techniques and maintaining the body through the use of large muscle movements, asymmetrical movements, and restorative relaxation | ND | 16 weeks | 3 | WC, FP, VF, LBM (InBody) |
| Liao et al., 2018 [48] | 56 | sarcopenic obesity | EX:66.7±4.5  CON:68.3±6.1 | EX:27.3±3.7  CON:29.2±3.6 | Resistance | whole body elastic band exercises; 3 sets with 10 reps at a rating of 13 BRPE | Supervised | 12 weeks | 3 | FP, MM (DEXA) |
| Maddalozzo et al., 2007 [49] | 69 | healthy | EX:52.3±3.3  CON:52.5±3.0 | ND | Resistance | resistance exercises; 3 sets with 8-12 reps at 60-75% of 1RM | Supervised | 52 weeks | 2 | LBM, FP (DEXA) |
| Malandish et al., 2020 [50] | 29 | vitamin D deficiency | EX:54.4±4.0  CON:54.0±3.3 | EX:28.6±4.2  CON:30.4±6.6 | Aerobic | 50-60 min at 65-70% of HRR | Supervised | 12 weeks | 3 | FP (DEXA) |
| Marcos-Pardo et al., 2019 [51] | 27 | healthy | EX:65-75  CON:65-75 | EX:27.6±4.4  CON:26.1±3.3 | Resistance | whole body exercises; 8-12 reps,  60-80% of 1RM | Supervised | 12 weeks | 3 | FP, LBM (InBody) |
| Marín-Cascales et al., 2015 [52] | 40 | healthy | EX:57.7±7.1  CON:62.4±5.1 | EX:29.3±3.9  CON:29.4±4.7 | Multi component exercise | small, reactive vertical jumps from 4x10 drop jumps to 6x10 +30-45 min at 50-60% of HRR | ND | 12 weeks | 3 | LBM (DEXA) |
| Marques et al., 2011 (a) [53] | 60 | healthy | EX:70.1±5.4  CON:68.2±5.7 | EX:28.4±3.7  CON:28.2±3.7 | Combined | 60-min multi-component exercises;  consisting of marching in place, stepping exercise at a speed of 120-125 beats per minute, resistance, balance, and agility | Supervised | 8 months  (32-week) | 2 | WC, FP, FFM (DEXA) |
| Marques et al., 2011 (b) [54] | 71 | healthy | EX_1_:70.3±5.5  EX_2_:67.3±5.2  CON:67.9±5.9 | EX_1_:27.5±3.8  EX_2_:28.8±4.6  CON:28.1±3.5 | EX_1_: aerobic EX_2_: resistance | A: 35-40 min at 50-85% of HRR  R: whole body exercises; 2 sets with 8-10 reps at 60-80% of 1RM | Supervised | 8 months (32 weeks) | 3 | WC, FP, FFM (DEXA) |
| Mazini Filho et al., 2018 [55] | 65 | healthy | EX:60-75  CON:60-75 | EX:27.0±4.5  CON:25.5±13.1 | Resistance | whole body exercises; 2-3 sets with 8-15 reps at a rating of 4-8 OMNI scale | ND | 12 weeks | 3 | WC |
| Monteiro et al., 2010 [56] | 93 | hormone therapy | EX:55.4±5.5  CON:59.2±7.4 | EX:27.3±4.3  CON:29.2±4.6 | Combined | A: 20-25 min at 50-85% of HRR  R: whole body exercises; 2 sets with 8-10 reps at 70-80% of 1RM | Supervised | 12 months | A: 3  R: 2 | MM (InBody) |
| Morrison et al., 1986 [57] | 32 | healthy | EX:51.1+2.8  CON:51.5+3.9 | ND | Aerobic | 40-min at 65-75% of HRR | Supervised | 8 months | 3 | FM, FP, LBM (Behnke and Wilmore technique) |
| Neves et al., 2017 [58] | 64 | healthy | ND | EX:27.1±3.7  CON:27.5±4.6 | Combined | whole body circuit resistance exercises using elastic bands plus 3 stations focused on balance, coordination, and agility + 18-30 minute walking | ND | 16 weeks | 3 | FM, FP, LBM (DEXA) |
| Nicholson et al., 2015 [59] | 57 | healthy | EX:66.0±4.1  CON:65.6±4.7 | EX:26.0±3.2  CON:24.5±2.9 | Resistance | whole body very high repetition resistance exercises | Supervised & Unsupervised | 6 months | 2 | FM, FFM (DEXA) |
| Nunes et al., 2016 [60] | 38 | healthy | EX_1_:62.7±6.3  EX_2_:60.7±9.1  CON:59.5±8.7 | EX_1_:27.9±0.7  EX_2_:28.1±8.7  CON:30.4±7.0 | Resistance | EX_1_: whole body exercises; 3 sets with 8-12 reps at 70% of 1RM  EX_2_: whole body exercises; 6 sets with 8-12 reps at 70% of 1RM | Supervised | 16 weeks | 3 | WC, FP (caliper) |
| Oh et al., 2021 [61] | 60 | knee osteoarthritis | EX:72.4±6.3  CON:71.1±5.4 | EX:24.8±2.5  CON:25.7±3.8 | Resistance | whole body chair and elastic band exercises | Supervised & Unsupervised | 5 months | 2-3 | FM, FP, MM (DEXA) |
| Oh et al., 2017 [62] | 80 | community dwelling | EX:74.9±6.5  CON:73.5±2.2 | EX:24.8±2.6  CON:25.0±2.6 | Resistance | whole body elastic band exercises; 2-3 sets with 10-20 reps at 10-20 RM | Supervised & Unsupervised | 18 weeks | 2 | FM, FFM (DEXA) |
| Orsatti et al., 2008 [63] | 50 | healthy | EX:57.8±8.0  CON:59.3±6.2 | EX:28.8±4.5  CON:27.6±5.1 | Resistance | whole body exercises; 1-3 sets with 8-15 reps at 40-80% of 1RM | Supervised | 16 weeks | 3 | WC, MM, FP (InBody) |
| Paolillo et al., 2014 [64] | 30 | healthy | EX:55.0±2.0  CON:55.0±2.0 | EX:27.0±4.0  CON:33.0±7.0 | Aerobic | 45-min at 85-90% of HR_max_ | Supervised | 6 months | 2 | FM, FP, LBM (InBody) |
| Park et al., 2017 [65] | 50 | sarcopenic obesity | EX:73.5±7.1  CON:74.7±5.1 | EX:27.0±1.4  CON:27.6±2.0 | Combined | A: 30-50 min at a rating of 13-17 BRPE  R: whole body elastic band exercises; 2-3 sets with 8-15 reps at 80% of 1RM | Supervised | 24 weeks | A: 5  R: 3 | WC |
| Park et al., 2021 [66] | 54 | early knee osteoarthritis | EX:66.9±4.6  CON:68.0±4.2 | ND | Resistance | Lower-body isometric exercises; at 60-80% of maximum tolerance or 10-15 of BRPE | ND | 8 weeks | 3 | FM, FP, MM (InBody) |
| Pospieszna et al., 2017 [67] | 39 | healthy | EX:62±3.79  CON:62±1.10 | EX:25.1±2.5  CON:26.0±0.8 | Aerobic | 50-min at 90% of ventilatory threshold intensity | Supervised | 12 weeks | 3 | FM, FFM (InBody) |
| Pu et al., 2001 [68] | 18 | chronic heart failure | EX:76.6±6.0  CON:76.6±6.3 | EX:24.7±3.6  CON:28.0±5.0 | Resistance | whole body exercises; 3 sets with 8 reps at 80% of 1RM | Supervised | 10 weeks | 3 | CSA (biopsies) |
| Rashti et al., 2019 [69] | 51 | overweight and obese | EX_1_:57.1±4.1  EX_2_:54.1±5.1  CON:54.1±5.1 | EX_1_:28.3±2.2  EX_2_:30.62±3.1  CON:29.9±4.2 | Combined | EX_1_: whole body exercises; 2-4 sets with 8-10 reps at 60-85% of 1RM + four sets of 4-min at 85-95% of HR_max_ by 4-min recovery at 65% of HR_max_  EX_2_: whole body exercises; 3-4 sets with 12-15 reps at 40-70% of 1RM + 50-min at 60-75% of HR_max_ | Supervised | 10 weeks | 3 | VF (MRI) |
| Raso et al., 2007 [70] | 42 | healthy | EX:67.0±5.0  CON:68.0±3.0 | EX:24.0±4.0  CON:26.0±4.0 | Resistance | whole body exercises; 3 sets with 12 reps at 60% of 1RM | Supervised | 12 months | 3 | FP (InBody) |
| Rezende et al., 2016 [71] | 44 | nonalcoholic fatty liver disease | EX:56.2±7.8  CON:54.5±8.9 | EX:34.1±4.4  CON:32.0±5.0 | Aerobic | 30-50 min at ventilatory anaerobic  threshold up to 10% below respiratory compensation point | Supervised | 24 weeks | 2 | FM, FP, MM (InBody) |
| Rhodes et al., 2000 [72] | 44 | healthy | EX:68.8±3.2  CON:68.2±3.5 | ND | Resistance | whole body exercises; 3 sets with 8 reps at 75% of 1RM | Supervised | 12 months | 3 | WC |
| Ribeiro et al., 2020 [73] | 43 | obese | EX:69.0±6.7  CON:67.1±4.1 | EX:30.7±4.6  CON:29.1±3.3 | Resistance | whole body exercises; 3 sets with 8-12 reps | Supervised | 8 weeks | 3 | FM, FP, FFM (DEXA) |
| Ribeiro et al., 2017 [74] | 76 | healthy | EX_1_:69.7±6.6  EX_2_:68.9±5.8  CON:66.8±4.2 | EX_1_:28.0±5.2  EX_2_:27.3±4.6  CON:26.3±4.6 | Resistance | EX_1_: whole body exercises; 3 sets with 8-12 reps with the same load in the three sets  EX_2_: whole body exercises; 3 sets with 8-12 reps with the load increasing and number of repetitions decreasing for each set | Supervised | 8 weeks | 3 | MM (DEXA) |
| Saarto et al., 2012 [75] | 269 | breast cancer | EX:58(48-68)  CON:58(46-68) | EX:27.2±4.4  CON:26.2±4.2 | Combined | The 45-min step aerobics and circuit training sessions were performed on alternate weeks at a rating of 11-16 BRPE | Supervised & Unsupervised | 12 months | 3-4 | FM, LBM (DEXA) |
| Santos et al., 2019 [76] | 27 | breast cancer survivors | EX:55.0±5.8  CON:54.3±5.2 | EX:28.0±5.0  CON:26.8±4.0 | Resistance | whole body exercises; 3 sets with 8-12 reps | Supervised | 8 weeks | 1 | FM, FP, LBM (DEXA) |
| Seo et al., 2021 [77] | 27 | sarcopenia | EX:70.3±5.4  CON:72.9±4.8 | EX:22.9±2.0  CON:22.4±1.5 | Resistance | whole body weight-based and elastic band; 3-5 sets with 6-15 reps at a rating of 4-8 OMNI Scale | Supervised | 16 weeks | 3 | FM, FP, FFM (DEXA), WC |
| Shaw et al., 2016 [78] | 37 | healthy | EX:60.4±5.3  CON:57.7±2.8 | EX:26.1±3.1  CON:24.5±3.9 | Resistance | whole body exercises; 3 sets with 8-10 reps at 67-85% of 1RM | ND | 6 weeks | 2 | FM, FP, LBM (caliper), WC |
| Sipilä and Suominen, 1995 [79] | 42 | healthy | EX_1_:76–78  EX_2_:76–78  CON:76-78 | ND | EX_1_: aerobic  EX_2_: resistance | EX_1_: 60-min at 50-80% of HRR  EX_2_: whole body exercises; 3-4 sets with 8-10 reps at 60-75% of 1RM | Supervised | 18 weeks | 2-3 | FP, LBM (InBody), CSA (DEXA) |
| Son et al., 2020 [80] | 25 | hypertension | EX:67.7±1.0  CON:67.4±1.1 | EX:26.4±1.1  CON:26.9±1.0 | Resistance | whole body resistant band exercises; at 60-80% of 1RM | Supervised | 12 weeks | 3 | FP, LBM (InBody) |
| Son et al., 2017 [81] | 20 | hypertension | EX:76.0±15.8  CON:74.7±6.3 | EX:22.8±2.2  CON:24.1±0.6 | Combined | A: 30-min at 40-70% of HRR  R: whole body resistant band exercises for 20 min | Supervised | 12 weeks | 3 | FP (InBody) |
| Souza et al., 2017 [82] | 45 | healthy | EX:67.3±4.3  CON:67.1±4.5 | EX:26.8±5.1  CON:26.6±4.7 | Resistance | whole body exercises; 3 sets with 10-15 reps | ND | 12 weeks | 3 | FP, MM (InBody) |
| Strandberg et al., 2019 [83] | 42 | healthy | EX:65-70  CON:65-70 | ND | Resistance | whole body exercises; 3 sets with 8-15 reps at 75-85% of 1RM | Supervised | 24 weeks | 2 | CSA (biopsies) |
| Taaffe et al., 1996 [84] | 36 | healthy | EX_1_:67.2±0.6  EX_2_:67.2±0.4  CON: 69.6±1.3 | EX_1_:24.9±1.0  EX_2_:24.3±0.5  CON:25.1±0.9 | Resistance | EX_1_: whole body exercises; 3 sets with 7 reps at 70-80% of 1RM  EX_2_: whole body exercises; 3 sets with 14 reps at 35-40% of 1RM | Supervised | 52 weeks | 3 | CSA (DEXA) |
| Taaffe et al., 2005 [85] | 40 | healthy | EX:50–57  CON:50-57 | EX:<33  CON:<33 | Resistance | lower body high-impact training included a circuit format included bounding, drop jumping, hopping and skipping + three or four resistance training exercises for the upper body | Supervised & Unsupervised | 1 year | 6 | FP, LBM, CSA (CT) |
| Taheri and Irandoust, 2018 [86] | 34 | sleep disorders | EX:60<age  CON:60<age | EX:27.2±09  CON:27.3±0.9 | Aerobic | 40-min at 50-60% of HR_max_ | ND | 2 months | 3 | FP (InBody) |
| Tan et al., 2018 [87] | 34 | type 2 diabetes | EX:63.0±2.3  CON:62.9±2.6 | EX:26.6±3.1  CON:26.5±3.2 | Aerobic | 20-40 min at maximal fat oxidation | Supervised | 12 weeks | 3 | WC, FM, FP, FFM (DEXA), VF (InBody), |
| Tomeleri et al., 2018 [88] | 53 | healthy | EX:72.1±6.3  CON:68.8±4.9 | EX:26.6±3.1  CON:27.3±4.2 | Resistance | whole body elastic band exercises; 3 sets with 8-15 reps | Supervised | 12 weeks | 3 | WC, FP, MM (DEXA), |
| Tsourlou et al., 2006 [89] | 24 | healthy | EX:69.3±6.6  CON:68.4±6.7 | EX:28.2±2.8  CON:29.3±3.5 | Water-based exercise | 25-min aerobic water-based exercise program at 65-80% of HR_max_ + whole body resistance exercises with specialized water-resistance equipment; 2-3 sets with 12-15 reps | Supervised | 24 weeks | 3 | FFM (InBody) |
| Tsutsumi et al., 1998 [90] | 36 | healthy | EX_1_:68.5±6.1  EX_2_:68.5±6.1  CON:68.5±6.1 | ND | Resistance | EX_1_: whole body elastic band exercises; 2 sets with 8-10 reps at 75-85% 1RM  EX_2_: whole body elastic band exercises; 2 sets with 14-16 reps at 55-65% 1RM | ND | 12 weeks | 3 | FP (caliper) |
| Urzi et al., 2019 [91] | 35 | living in a nursing home | EX:84.4±7.7  CON:88.9±5.3 | EX:28.0±5.5  CON:29.1±5.1 | Resistance | whole body elastic band exercises; at a rating of 12-14 BRPE | Supervised | 12 weeks | 3 | MM (ND) |
| van Gemert et al., 2015 [92] | 146 | overweight and obese | EX:59.5±4.9  CON:60.0±4.9 | EX:29.0±2.9  CON:29.5±2.6 | Combined | A: 60-min at 60-95% of HRR  R: strength training | Supervised | 16 weeks | 4 | FP (DEXA) |
| Vélez-Toral et al., 2017 [93] | 166 | healthy | EX:56.2±4.1  CON:55.7±4.0 | EX:27.0±3.7  CON:27.3±4.9 | Combined | aerobic cardiorespiratory fitness, muscle resistance and other fitness functions | Supervised | 20 weeks | 3 | WC |
| Verschueren et al., 2004 [94] | 46 | healthy | EX:63.9±3.8  CON:64.2±3.1 | EX:27.4±3.5  CON:26.5±5.8 | Resistance | Lower body exercise; 2-3 sets with 10-15 reps | ND | 24 weeks | 3 | FM, FP (DEXA) |
| von Stengel et al., 2012 [95] | 101 | Older adults | EX:68.6±3.0  CON:68.1±2.7 | EX:26.2±4.2  CON:27.5±5.0 | Combined | A: 20-min at 70-80% of HR_max_  R: 35-min strength training | Supervised | 18 months | 2 | FP, LBM (DEXA) |
| Wen et al., 2017 [96] | 48 | low bone mass | EX:57.5±3.5  CON:58.8±3.2 | EX:21.7±2.9  CON:22.3±1.9 | Aerobic | 30-35 min step aerobics at 75-85% of heart rate reserve | Supervised | 10 weeks | 3 | FP (DEXA) |
| Son and Park, 2021 [97] | 42 | obese | EX:68.2±1.6  CON:68.2±1.4 | EX:26.7±3.2  CON:27.1±1.4 | Resistance | Whole body elastic band exercises; 2-4 sets with 5-20 reps at 40-70% of 1RM | Supervised | 12 weeks | 3 | FP, LBM (InBody), WC |
| Wong et al., 2018 [98] | 44 | stage 2 hypertension | EX:59.0±4.5  CON:59.0±4.6 | EX:24.2±3.6  CON:23.8±3.7 | Aerobic | 2-5 ×12 sets of 192 climbing at a rating of 11-13 BRPE by 5 min recovery | Supervised | 12 weeks | 4 | FM, FFM (InBody) |
| Wong et al., 2018 [99] | 100 | stage 2 hypertension | EX:74.0±4,0  CON:73.0±4.0 | EX:26.0±2.8  CON: 26.9±2.9 | Water-based exercise | 25-45 min at 60-75% of HR_max_ | Supervised | 20 weeks | 3-4 | FM, FP, FFM (InBody) |
| Yoo et al., 2010 [100] | 28 | community-dwelling | EX:70.9±2.7  CON:71.1±2.7 | EX:26.6±2.9  CON:25.4±3.0 | Aerobic | 45-min at 60% of HRR | Supervised | 12 weeks | 3 | FM, LBM (DEXA) |
| Yoon et al., 2017 [101] | 58 | mild cognitive impairment | EX_1_:75.0±0.9  EX_2_:76.0±1.3  CON:78.0±1.0 | EX_1_: 25.5±2.5  EX_2_: 23.9±3.7  CON: 22.9±1.8 | Resistance | EX_1_: elastic band exercises; 2-3 sets with 12-15 reps at a rating of 12-13 BRPE  EX_2_: elastic band exercises; 2-3 sets with 8-10 reps at a rating of 15-16 BRPE | Supervised | 12 weeks | 2 | FP, MM (InBody) |
| **Abbreviations:** body fat percentage (FP), fat mass (FM), muscle mass (MM), muscle fiber cross-sectional area (CSA), lean body mass (LBM), fat free mass (FFM), waist circumference (WC), visceral fat (VF), maximal/peak heart rate (HR_max/peak_), one repetition maximum (1RM), Borg rating of perceived exertion (BRPE), repetitions (reps), computed tomography scan (CT), Dual-energy X-ray absorptiometry (DEXA), not-described (ND) | | | | | | | | | | |

**Supplementary Table 2.** Risk of bias assessment

| **Authors & Year** | **Criteria 1** | **Criteria 2** | **Criteria 3** | **Criteria 4** | **Criteria 5** | **Criteria 6** | **Criteria 7** | **Criteria 8** | **Criteria 9** | **total** |
| --- | --- | --- | --- | --- | --- | --- | --- | --- | --- | --- |
| Ades et al. 2005 [1] | ✓ | ✓ | x | ✓ | x | ✓ | x | ✓ | ✓ | 6 |
| Aragão et al. 2014 [2] | ✓ | ✓ | ? | ✓ | x | x | x | ✓ | ✓ | 5 |
| Bea et al. 2010 [3] | ✓ | ✓ | x | ✓ | x | ✓ | x | ✓ | ✓ | 6 |
| Blain et al. 2017 [4] | ✓ | ✓ | ✓ | ✓ | ✓ | x | ✓ | ✓ | ✓ | 8 |
| Boutcher et al. 2019 [5] | ✓ | ✓ | x | ✓ | ✓ | ✓ | x | ✓ | ✓ | 7 |
| Campa et al. 2018 [6] | ✓ | ✓ | ✓ | ✓ | x | ✓ | x | ✓ | ✓ | 7 |
| Cao et al. 2019 [7] | ? | ✓ | ? | ✓ | x | ✓ | x | ✓ | ✓ | 5 |
| Cao et al. 2009 [8] | x | ✓ | x | x | x | ✓ | x | ✓ | ✓ | 4 |
| Cavalcante et al. 2018 [9] | ✓ | ✓ | ✓ | ✓ | ✓ | ✓ | x | ✓ | ✓ | 8 |
| Charette et al. 1991 [10] | ✓ | ✓ | x | ✓ | x | ✓ | x | ✓ | ✓ | 6 |
| Chen et al. 2018 [11] | ✓ | ✓ | x | ✓ | x | ✓ | x | ✓ | ✓ | 6 |
| Coelho-Júnior et al. 2019 [12] | ✓ | ✓ | ✓ | ✓ | x | x | x | ✓ | ✓ | 6 |
| Conceição et al. 2013 [13] | ✓ | ✓ | x | ✓ | x | ✓ | x | ✓ | ✓ | 6 |
| Correa et al. 2014 [14] | ✓ | ✓ | x | ✓ | x | ✓ | x | ✓ | ✓ | 6 |
| Cunha et al. 2018 [15] | ✓ | ✓ | ✓ | ✓ | x | ✓ | x | ✓ | ✓ | 7 |
| Cunha et al. 2021 [16] | ✓ | ✓ | ✓ | ✓ | x | ✓ | x | ✓ | ✓ | 7 |
| De Vito et al. 1999 [17] | ✓ | ✓ | x | ✓ | x | ✓ | x | ✓ | ✓ | 6 |
| do Nascimento et al. 2018 [18] | ✓ | ✓ | x | ✓ | ✓ | x | x | ✓ | ✓ | 6 |
| Dobek et al. 2014 [19] | ✓ | ✓ | x | ✓ | ✓ | x | x | ✓ | ✓ | 6 |
| dos Santos et al. 2020 [20] | ✓ | ✓ | x | ✓ | ✓ | ✓ | ✓ | ✓ | ✓ | 8 |
| Duff et al. 2017 [21] | ✓ | ✓ | ✓ | ✓ | ✓ | x | ✓ | ✓ | ✓ | 8 |
| Elsangedy et al. 2021 [22] | ✓ | ✓ | x | ✓ | x | ✓ | x | ✓ | ✓ | 6 |
| Englund et al. 2005 [23] | ✓ | ✓ | x | ✓ | x | ✓ | x | ✓ | ✓ | 6 |
| Faramarzi et al. 2018 [24] | ✓ | ✓ | x | ✓ | x | ✓ | x | ✓ | ✓ | 6 |
| Félix-Soriano et al. 2021 [25] | ✓ | ✓ | ✓ | ✓ | x | x | x | ✓ | ✓ | 6 |
| Figueroa et al. 2003 [26] | ✓ | ✓ | x | ✓ | x | ✓ | x | ✓ | ✓ | 6 |
| Figueroa et al. 2011 [27] | ✓ | ✓ | x | ✓ | x | ✓ | x | ✓ | ✓ | 6 |
| Flynn et al. 1999 [28] | ✓ | ✓ | x | ✓ | x | ✓ | x | ✓ | ✓ | 6 |
| Fourie et al. 2013 [29] | ✓ | ✓ | x | ✓ | x | ✓ | x | ✓ | ✓ | 6 |
| Friedenreich et al. 2011 [30] | ? | ✓ | x | ? | x | ✓ | x | ✓ | ✓ | 4 |
| Fritz et al. 2018 [31] | ✓ | ✓ | ? | ✓ | x | x | x | ✓ | ✓ | 5 |
| Frontera et al. 2003 [32] | ? | ✓ | ? | ✓ | ✓ | ✓ | x | ✓ | ✓ | 6 |
| Gadelha et al. 2016 [33] | ✓ | ✓ | ? | ? | x | ✓ | x | ✓ | ✓ | 5 |
| Gerage et al. 2013 [34] | ✓ | ✓ | x | x | ✓ | ✓ | x | ✓ | ✓ | 6 |
| Cássio et al. 2014 [35] | ✓ | ✓ | x | ✓ | x | ✓ | x | ✓ | ✓ | 6 |
| González-Ravé et al. 2020 [36] | ✓ | ✓ | x | ✓ | x | ✓ | x | ✓ | ✓ | 6 |
| Grove and Londeree. 1992 [37] | ✓ | ✓ | x | ? | x | ✓ | x | ✓ | ✓ | 5 |
| Gualano et al. 2014 [38] | ✓ | ✓ | ✓ | ? | x | x | x | ✓ | ✓ | 5 |
| Ha et al. 2021 [39] | ✓ | ✓ | ✓ | ✓ | x | ✓ | x | ✓ | ✓ | 7 |
| Holsgaard-Larsen et al. 2011 [40] | ✓ | ✓ | x | ✓ | x | ✓ | x | ✓ | ✓ | 6 |
| Hoseini et al. 2020 [41] | ✓ | ✓ | x | ? | x | ✓ | x | ✓ | ✓ | 5 |
| Im et al. 2019 [42] | ✓ | ✓ | x | ✓ | x | ✓ | x | ✓ | ✓ | 6 |
| Jang and Park. 2021 [43] | ✓ | ✓ | x | ? | x | ✓ | x | ✓ | ✓ | 5 |
| Janzen et al. 2006 [44] | ✓ | ✓ | ✓ | ✓ | x | ✓ | x | ✓ | ✓ | 7 |
| Kallinen et al. 2002 [45] | ✓ | ✓ | x | ✓ | x | x | x | ✓ | ✓ | 5 |
| Kim et al. 2016 [46] | ✓ | ✓ | x | ✓ | x | ✓ | x | ✓ | ✓ | 6 |
| Lee et al. 2012 [47] | ✓ | ✓ | x | ✓ | x | ✓ | x | ✓ | ✓ | 6 |
| Liao et al. 2018 [48] | ✓ | ✓ | x | ✓ | x | x | x | ✓ | ✓ | 5 |
| Maddalozzo et al. 2007 [49] | ✓ | ✓ | x | ✓ | x | ✓ | x | ✓ | ✓ | 6 |
| Malandish et al. 2020 [50] | ✓ | ✓ | x | ✓ | x | ✓ | x | ✓ | ✓ | 6 |
| Marcos-Pardo et al. 2019 [51] | ✓ | ✓ | x | ? | x | x | x | ✓ | ✓ | 4 |
| Marín-Cascales et al. 2015 [52] | ✓ | ✓ | x | ✓ | x | x | x | ✓ | ✓ | 5 |
| Marques et al. 2011 (a) [53] | ✓ | ✓ | ✓ | ✓ | x | x | ✓ | ✓ | ✓ | 7 |
| Marques et al. 2011 (b) [54] | ✓ | ✓ | ✓ | ✓ | ✓ | x | ✓ | ✓ | ✓ | 8 |
| Mazini Filho et al. 2018 [55] | ✓ | ✓ | x | ? | x | ✓ | x | ✓ | ✓ | 5 |
| Monteiro et al. 2010 [56] | ✓ | ✓ | x | ✓ | x | ✓ | ✓ | ✓ | ✓ | 7 |
| Morrison et al. 1986 [57] | ✓ | ✓ | x | ✓ | x | x | x | ✓ | ✓ | 5 |
| Neves et al. 2017 [58] | ✓ | ✓ | x | ✓ | ✓ | x | x | ✓ | ✓ | 6 |
| Nicholson et al. 2015 [59] | ✓ | ✓ | x | ✓ | ✓ | ✓ | ✓ | ✓ | ✓ | 8 |
| Nunes et al. 2016 [60] | ✓ | ✓ | x | ✓ | x | x | x | ✓ | ✓ | 5 |
| Oh et al. 2021 [61] | ✓ | ✓ | x | ✓ | x | x | x | ✓ | ✓ | 5 |
| Oh et al. 2017 [62] | ✓ | ✓ | x | ✓ | x | x | x | ✓ | ✓ | 5 |
| Orsatti et al. 2008 [63] | ✓ | ✓ | x | ✓ | x | ✓ | x | ✓ | ✓ | 6 |
| Paolillo et al. 2014 [64] | ✓ | ✓ | x | x | x | x | x | ✓ | ✓ | 4 |
| Park et al. 2017 [65] | ✓ | ✓ | ? | ✓ | x | ✓ | x | ✓ | ✓ | 6 |
| Park et al. 2021 [66] | ✓ | ✓ | x | ✓ | x | ✓ | x | ✓ | ✓ | 6 |
| Pospieszna et al. 2017 [67] | ✓ | ✓ | x | x | x | ✓ | x | ✓ | ✓ | 5 |
| Pu et al. 2001 [68] | ✓ | ✓ | ✓ | ✓ | ✓ | ✓ | ✓ | ✓ | ✓ | 9 |
| Rashti et al. 2019 [69] | ✓ | ✓ | x | ✓ | x | x | x | ✓ | ✓ | 5 |
| Raso et al. 2007 [70] | ✓ | ✓ | x | ✓ | x | ? | x | ✓ | ✓ | 5 |
| Rezende et al. 2016 [71] | ✓ | ✓ | x | ✓ | x | ✓ | x | ✓ | ✓ | 6 |
| Rhodes et al. 2000 [72] | ✓ | ✓ | x | ✓ | x | ✓ | x | ✓ | ✓ | 6 |
| Ribeiro et al. 2020 [73] | ✓ | ✓ | ? | ✓ | ✓ | x | x | ✓ | ✓ | 6 |
| Ribeiro et al. 2017 [74] | ✓ | ✓ | ✓ | ✓ | ✓ | ✓ | ✓ | ✓ | ✓ | 9 |
| Saarto et al. 2012 [75] | ✓ | ✓ | ✓ | ? | ✓ | ✓ | ✓ | ✓ | ✓ | 8 |
| Santos et al. 2019 [76] | ✓ | ✓ | x | ✓ | x | ✓ | x | ✓ | ✓ | 6 |
| Seo et al. 2021 [77] | ✓ | ✓ | x | ✓ | ? | x | x | ✓ | ✓ | 5 |
| Shaw et al. 2016 [78] | ✓ | ✓ | ✓ | x | ? | ✓ | x | ✓ | ✓ | 6 |
| Sipilä and Suominen. 1995 [79] | ✓ | ✓ | x | ✓ | x | x | x | ✓ | ✓ | 5 |
| Son et al. 2020 [80] | ✓ | ✓ | ✓ | ✓ | ✓ | ✓ | ✓ | ✓ | ✓ | 9 |
| Son et al. 2017 [81] | ✓ | ✓ | x | ✓ | ✓ | ✓ | x | ✓ | ✓ | 7 |
| Souza et al. 2017 [82] | ✓ | ✓ | x | ✓ | ✓ | ✓ | x | ✓ | ✓ | 7 |
| Strandberg et al. 2019 [83] | ✓ | ✓ | x | ✓ | x | ✓ | x | ✓ | ✓ | 6 |
| Taaffe et al. 1996 [84] | ? | ✓ | x | ✓ | x | x | x | ✓ | ✓ | 4 |
| Taaffe et al. 2005 [85] | ✓ | ✓ | ? | ✓ | x | x | x | ✓ | ✓ | 5 |
| Taheri and Irandoust. 2018 [86] | ✓ | ✓ | x | ✓ | x | ✓ | x | ✓ | ✓ | 6 |
| Tan et al. 2018 [87] | ✓ | ✓ | x | ✓ | x | ✓ | x | ✓ | ✓ | 6 |
| Tomeleri et al. 2018 [88] | ✓ | ✓ | ✓ | ✓ | x | ✓ | x | ✓ | ✓ | 7 |
| Tsourlou et al. 2006 [89] | ✓ | ✓ | x | ✓ | x | ✓ | x | ✓ | ✓ | 6 |
| Tsutsumi et al. 1998 [90] | x | ✓ | x | ✓ | x | ✓ | x | ✓ | ✓ | 5 |
| Urzi et al. 2019 [91] | ✓ | ✓ | x | ✓ | x | x | x | ✓ | ✓ | 5 |
| van Gemert et al. 2015 [92] | ✓ | ✓ | x | ✓ | x | ✓ | ✓ | ✓ | ✓ | 7 |
| Vélez-Toral et al. 2017 [93] | ✓ | ✓ | ✓ | ✓ | ✓ | ✓ | x | ✓ | ✓ | 8 |
| Verschueren et al. 2004 [94] | ✓ | ✓ | ✓ | ✓ | x | ✓ | x | ✓ | ✓ | 7 |
| von Stengel et al. 2012 [95] | ✓ | ✓ | x | ✓ | ✓ | ✓ | ✓ | ✓ | ✓ | 8 |
| Wen et al. 2017 [96] | ✓ | ✓ | x | ✓ | x | ✓ | x | ✓ | ✓ | 6 |
| Son and Park. 2021 [97] | ✓ | ✓ | ✓ | ✓ | x | x | x | ✓ | ✓ | 7 |
| Wong et al. 2018 [98] | ✓ | ✓ | x | ✓ | x | ✓ | x | ✓ | ✓ | 6 |
| Wong et al. 2018 [99] | ✓ | ✓ | x | ? | ✓ | ✓ | x | ✓ | ✓ | 6 |
| Yoo et al. 2010 [100] | ✓ | ✓ | x | ✓ | x | x | x | ✓ | ✓ | 5 |
| Yoon et al. 2017 [101] | ✓ | ✓ | x | ? | x | x | x | ✓ | ✓ | 4 |

(1) Eligibility Criteria specified, (2) Random allocation of participants, (3) Allocation concealed, (4) Groups similar at baseline, (5) Assessors blinded, (6) Outcome measures assessed in 85% of participants, (7) Intention to treat analysis, (8) Reporting of between group statistical comparison, (9) Point measures and measures of variability reported for main effects. ‘low (✓), ‘high (x) and unclear (?)

Supplementary Table 3. Summary of subgroup analyses

|  |  | | Moderators | N | SMD (95% CI) | P-value | P-heterogeneity |
| --- | --- | --- | --- | --- | --- | --- | --- |
| **Muscle mass** | | Age (year) | middle<65 | 25 | 0.26 (0.19 to 0.43) | 0.001 | 0.75 |
|  |  |  | elderly≥65 | 9 | 0.26 (0.05 to 0.46) | 0.01 | 0.99 |
|  |  | Type of CT | Medium-term | 19 | 0.26 (0.09 to 0.44) | 0.002 | 0.99 |
|  |  |  | Long-term | 7 | 0.26 (0.06 to 0.45) | 0.008 | 0.68 |
|  |  | Sex | aerobic | 1 | - | - | - |
|  |  |  | Combined | 5 | 0.26 (0.04 to 0.48) | 0.02 | 0.99 |
|  |  |  | Resistance | 20 | 0.07 (0.11 to 0.44) | 0.001 | 0.98 |
| **Muscle and fiber CSA** | | Age | middle<65 | 2 | - | - | - |
|  |  |  | elderly≥65 | 13 | 0.59 (0.31 to 0.87) | 0.001 | 0.53 |
|  |  | Intervention duration | Medium-term | 6 | 0.64 (0.09 to 1.19) | 0.02 | 0.12 |
|  |  |  | Long-term | 9 | 0.44 (0.13 to 0.75) | 0.005 | 0.81 |
|  |  | Type of exercise | aerobic | 2 | - | - | - |
|  |  |  | Combined | - | - | - | - |
|  |  |  | Resistance | 13 | 0.57 (0.30 to 0.84) | 0.001 | 0.48 |
| **FFM** | | Age | middle<65 | 22 | 0.71 (0.41 to 1.00) | 0.001 | 0.11 |
|  |  |  | elderly≥65 | 33 | 0. (0.57 to 1.14) | 0.001 | 0.96 |
|  |  | Intervention duration | Medium-term | 25 | 0.83 (0.41 to 1.25) | 0.001 | 1.00 |
|  |  |  | Long-term | 31 | 0.79 (0.50 to 1.08) | 0.001 | 0.04 |
|  |  | Type of exercise | aerobic | 11 | 0.10 (-0.19 to 0.40) | 0.48 | 0.97 |
|  |  |  | Combined | 7 | 0.68 (0.31 to 1.06) | 0.001 | 0.37 |
|  |  |  | Resistance | 29 | 0.90 (0.68 to 1.11) | 0.001 | 0.99 |
| **Fat mass** | | Age | middle<65 | 19 | -1.15 (-1.74 to -0.55) | 0.001 | 0.007 |
|  |  |  | elderly≥65 | 23 | -1.11 (-2.35 to 0.13) | 0.08 | 0.001 |
|  |  | Intervention duration | Medium-term | 25 | -1.17 (-1.91 to -0.43) | 0.002 | 0.99 |
|  |  |  | Long-term | 18 | -1.24 (-2.31 to -0.17) | 0.02 | 0.001 |
|  | | Type of exercise | aerobic | 9 | -1.94 (-2.46 to -1.41) | 0.001 | 0.83 |
|  |  |  | Combined | 6 | 0.81 (-1.91 to 0.27) | 0.14 | 0.88 |
|  |  |  | Resistance | 24 | -0.42 (-0.86 to 0.01) | 0.06 | 1.00 |
| **Body fat percentage** | | Age | middle<65 | 36 | -1.92 (-2.56 to -1.28) | 0.001 | 0.001 |
|  |  |  | elderly≥65 | 48 | -1.76 (-2.77 to -0.75) | 0.001 | 0.001 |
|  |  | Intervention duration | Medium-term | 46 | -1.79 (-2.14 to -1.43) | 0.001 | 0.85 |
|  |  |  | Long-term | 39 | -1.82 (-2.84 to -0.81) | 0.001 | 0.001 |
|  |  | Type of exercise | aerobic | 15 | -1.68 (-2.39 to -0.97) | 0.001 | 0.13 |
|  |  |  | Combined | 14 | -2.24 (-3.08 to -1.39) | 0.001 | 0.003 |
|  |  |  | Resistance | 47 | -1.20 (-1.59 to -0.80) | 0.001 | 0.32 |
| **Waist circumference** | | Age | middle<65 | 17 | -1.42 (-2.25 to -0.59) | 0.001 | 0.34 |
|  |  |  | elderly≥65 | 9 | -1.50 (-2.94 to -0.06) | 0.04 | 0.99 |
|  |  | Intervention duration | Medium-term | 12 | -2.69 (-4.31 to -1.06) | 0.001 | 0.99 |
|  |  |  | Long-term | 14 | -1.18 (-1.93 to -0.43) | 0.002 | 0.37 |
|  |  | Type of exercise | aerobic | 3 | -2.30 (-3.36 to -1.24) | 0.001 | 0.97 |
|  |  |  | Combined | 5 | -1.66 (-3.21 to -0.10) | 0.03 | 0.53 |
|  |  |  | Resistance | 15 | -0.45 (-1.54 to 0.62) | 0.40 | 0.78 |

**References**

1. Ades, P.A., et al., Resistance training increases total daily energy expenditure in disabled older women with coronary heart disease. J Appl Physiol (1985), 2005. **98**(4): p. 1280-5.

2. Aragão, F.R., et al., Effects of a 12-month multi-component exercise program on the body composition of postmenopausal women. Climacteric, 2014. **17**(2): p. 155-63.

3. Bea, J.W., et al., Lifestyle modifies the relationship between body composition and adrenergic receptor genetic polymorphisms, ADRB2, ADRB3 and ADRA2B: a secondary analysis of a randomized controlled trial of physical activity among postmenopausal women. Behav Genet, 2010. **40**(5): p. 649-59.

4. Blain, H., et al., Effect of a 6-month brisk walking program on walking endurance in sedentary and physically deconditioned women aged 60 or older: A randomized trial. The journal of nutrition, health & aging, 2017. **21**(10): p. 1183-1189.

5. Boutcher, Y.N., et al., The effect of sprint interval training on body composition of postmenopausal women. Medicine & Science in Sports & Exercise, 2019. **51**(7): p. 1413-1419.

6. Campa, F., A.M. Silva, and S. Toselli, Changes in phase angle and handgrip strength induced by suspension training in older women. International journal of sports medicine, 2018. **39**(06): p. 442-449.

7. Cao, L., et al., Exercise training at maximal fat oxidation intensity for overweight or obese older women: A randomized study. Journal of Sports Science & Medicine, 2019. **18**(3): p. 413.

8. Cao, Z.-B., I. Tabata, and H. Nishizono, Good maintenance of physical benefits in a 12-month exercise and nutritional intervention by voluntary, home-based exercise: a 6-month follow-up of a randomized controlled trial. Journal of bone and mineral metabolism, 2009. **27**(2): p. 182-189.

9. Cavalcante, E.F., et al., Effects of different resistance training frequencies on fat in overweight/obese older women. International journal of sports medicine, 2018. **39**(07): p. 527-534.

10. Charette, S., et al., Muscle hypertrophy response to resistance training in older women. Journal of applied Physiology, 1991. **70**(5): p. 1912-1916.

11. Chen, H.-T., et al., Effects of 8-week kettlebell training on body composition, muscle strength, pulmonary function, and chronic low-grade inflammation in elderly women with sarcopenia. Experimental gerontology, 2018. **112**: p. 112-118.

12. Coelho-Júnior, H.J., et al., Periodized and non-periodized resistance training programs on body composition and physical function of older women. Experimental Gerontology, 2019. **121**: p. 10-18.

13. Conceição, M.S., et al., Sixteen weeks of resistance training can decrease the risk of metabolic syndrome in healthy postmenopausal women. Clinical interventions in aging, 2013. **8**: p. 1221.

14. Correa, C.S., et al., Effects of high and low volume of strength training on muscle strength, muscle volume and lipid profile in postmenopausal women. Journal of Exercise Science & Fitness, 2014. **12**(2): p. 62-67.

15. Cunha, P.M., et al., The effects of resistance training volume on osteosarcopenic obesity in older women. Journal of Sports Sciences, 2018. **36**(14): p. 1564-1571.

16. Cunha, P.M., et al., Comparision of low and high volume of resistance training on body fat and blood biomarkers in untrained older women: A randomized clinical trial. The Journal of Strength & Conditioning Research, 2021. **35**(1): p. 1-8.

17. De Vito, G., et al., Effects of a low-intensity conditioning programme on VO2max and maximal instantaneous peak power in elderly women. Eur J Appl Physiol Occup Physiol, 1999. **80**(3): p. 227-32.

18. do Nascimento, M.A., et al., Resistance training with dietary intake maintenance increases strength without altering body composition in older women. J Sports Med Phys Fitness, 2018. **58**(4): p. 457-464.

19. Dobek, J., et al., Musculoskeletal changes after 1 year of exercise in older breast cancer survivors. J Cancer Surviv, 2014. **8**(2): p. 304-11.

20. Dos Santos, L., et al., Effects of Pyramid Resistance-Training System with Different Repetition Zones on Cardiovascular Risk Factors in Older Women: A Randomized Controlled Trial. Int J Environ Res Public Health, 2020. **17**(17).

21. Duff, W.R., et al., Effects of Ibuprofen and Resistance Training on Bone and Muscle: A Randomized Controlled Trial in Older Women. Med Sci Sports Exerc, 2017. **49**(4): p. 633-640.

22. Elsangedy, H.M., et al., Effects of Self-selected Resistance Training on Physical Fitness and Psychophysiological Responses in Physically Inactive Older Women: A Randomized Controlled Study. Percept Mot Skills, 2021. **128**(1): p. 467-491.

23. Englund, U., et al., A 1-year combined weight-bearing training program is beneficial for bone mineral density and neuromuscular function in older women. Osteoporos Int, 2005. **16**(9): p. 1117-23.

24. Faramarzi, M., L. Bagheri, and E. Banitalebi, Effect of sequence order of combined strength and endurance training on new adiposity indices in overweight elderly women. Isokinetics and Exercise Science, 2018. **26**: p. 105-113.

25. Félix-Soriano, E., et al., Effects of DHA-Rich n-3 Fatty Acid Supplementation and/or Resistance Training on Body Composition and Cardiometabolic Biomarkers in Overweight and Obese Post-Menopausal Women. Nutrients, 2021. **13**(7): p. 2465.

26. Figueroa, A., et al., Effects of exercise training and hormone replacement therapy on lean and fat mass in postmenopausal women. J Gerontol A Biol Sci Med Sci, 2003. **58**(3): p. 266-70.

27. Figueroa, A., et al., Combined resistance and endurance exercise training improves arterial stiffness, blood pressure, and muscle strength in postmenopausal women. Menopause, 2011. **18**(9): p. 980-4.

28. Flynn, M.G., et al., Effects of resistance training on selected indexes of immune function in elderly women. J Appl Physiol (1985), 1999. **86**(6): p. 1905-13.

29. Fourie, M., et al., Effects of a mat Pilates programme on body composition in elderly women. West Indian Med J, 2013. **62**(6): p. 524-8.

30. Friedenreich, C.M., et al., Adiposity changes after a 1-year aerobic exercise intervention among postmenopausal women: a randomized controlled trial. Int J Obes (Lond), 2011. **35**(3): p. 427-35.

31. Fritz, N.B., et al., Positive Effects of a Short-Term Intense Elastic Resistance Training Program on Body Composition and Physical Functioning in Overweight Older Women. Biol Res Nurs, 2018. **20**(3): p. 321-334.

32. Frontera, W.R., et al., Strength training in older women: early and late changes in whole muscle and single cells. Muscle Nerve, 2003. **28**(5): p. 601-8.

33. Gadelha, A.B., et al., Effects of resistance training on sarcopenic obesity index in older women: A randomized controlled trial. Arch Gerontol Geriatr, 2016. **65**: p. 168-73.

34. Gerage, A.M., et al., Cardiovascular adaptations to resistance training in elderly postmenopausal women. Int J Sports Med, 2013. **34**(9): p. 806-13.

35. Goncalves, C.G.S., et al., Functional and physiological effects of a 12-week programme of resistance training in elderly hypertensive women : original research article. International SportMed Journal, 2014. **15**(1): p. 50-61.

36. González-Ravé, J.M., et al., Strength Improvements of Different 10-Week Multicomponent Exercise Programs in Elderly Women. Front Public Health, 2020. **8**: p. 130.

37. Grove, K.A. and B.R. Londeree, Bone density in postmenopausal women: high impact vs low impact exercise. Med Sci Sports Exerc, 1992. **24**(11): p. 1190-4.

38. Gualano, B., et al., Creatine supplementation and resistance training in vulnerable older women: a randomized double-blind placebo-controlled clinical trial. Exp Gerontol, 2014. **53**: p. 7-15.

39. Ha, M.S., et al., Exercise training and burdock root (Arctium lappa L.) extract independently improve abdominal obesity and sex hormones in elderly women with metabolic syndrome. Sci Rep, 2021. **11**(1): p. 5175.

40. Holsgaard-Larsen, A., et al., Stair-ascent performance in elderly women: effect of explosive strength training. J Aging Phys Act, 2011. **19**(2): p. 117-36.

41. Hoseini, Z., N. Behpour, and R. Hoseini, Co-treatment with Vitamin D Supplementation and Aerobic Training in Elderly Women with Vit D Deficiency and NAFLD: A Single-blind Controlled Trial. 2020. **20**(2): p. e96437.

42. Im, J.Y., H.S. Bang, and D.Y. Seo, The Effects of 12 Weeks of a Combined Exercise Program on Physical Function and Hormonal Status in Elderly Korean Women. Int J Environ Res Public Health, 2019. **16**(21).

43. Jang, E.M. and S.H. Park, Effects of Neuromuscular Electrical Stimulation Combined with Exercises versus an Exercise Program on the Physical Characteristics and Functions of the Elderly: A Randomized Controlled Trial. Int J Environ Res Public Health, 2021. **18**(5).

44. Janzen, C.L., P.D. Chilibeck, and K.S. Davison, The effect of unilateral and bilateral strength training on the bilateral deficit and lean tissue mass in post-menopausal women. Eur J Appl Physiol, 2006. **97**(3): p. 253-60.

45. Kallinen, M., et al., Improving cardiovascular fitness by strength or endurance training in women aged 76-78 years. A population-based, randomized controlled trial. Age Ageing, 2002. **31**(4): p. 247-54.

46. Kim, H., et al., Exercise and Nutritional Supplementation on Community-Dwelling Elderly Japanese Women With Sarcopenic Obesity: A Randomized Controlled Trial. J Am Med Dir Assoc, 2016. **17**(11): p. 1011-1019.

47. Lee, J.A., J.W. Kim, and D.Y. Kim, Effects of yoga exercise on serum adiponectin and metabolic syndrome factors in obese postmenopausal women. Menopause, 2012. **19**(3): p. 296-301.

48. Liao, C.D., et al., Effects of elastic band exercise on lean mass and physical capacity in older women with sarcopenic obesity: A randomized controlled trial. Sci Rep, 2018. **8**(1): p. 2317.

49. Maddalozzo, G.F., et al., The effects of hormone replacement therapy and resistance training on spine bone mineral density in early postmenopausal women. Bone, 2007. **40**(5): p. 1244-51.

50. Malandish, A., et al., The effect of moderate-intensity aerobic training on pulmonary function and estrogen receptor-alpha gene in postmenopausal women with vitamin D deficiency: A randomized control trial. Respir Physiol Neurobiol, 2020. **281**: p. 103510.

51. Marcos-Pardo, P.J., et al., Effects of a moderate-to-high intensity resistance circuit training on fat mass, functional capacity, muscular strength, and quality of life in elderly: A randomized controlled trial. Sci Rep, 2019. **9**(1): p. 7830.

52. Marín-Cascales, E., et al., Effect of 12 Weeks of Whole-Body Vibration Versus Multi-Component Training in Post-Menopausal Women. Rejuvenation Res, 2015. **18**(6): p. 508-16.

53. Marques, E.A., et al., Multicomponent training program with weight-bearing exercises elicits favorable bone density, muscle strength, and balance adaptations in older women. Calcif Tissue Int, 2011. **88**(2): p. 117-29.

54. Marques, E.A., et al., Effects of resistance and aerobic exercise on physical function, bone mineral density, OPG and RANKL in older women. Exp Gerontol, 2011. **46**(7): p. 524-32.

55. Mazini Filho, M.L., et al., Circuit strength training improves muscle strength, functional performance and anthropometric indicators in sedentary elderly women. J Sports Med Phys Fitness, 2018. **58**(7-8): p. 1029-1036.

56. Monteiro, M.A., et al., Exercise effects in plantar pressure of postmenopausal women. Menopause, 2010. **17**(5): p. 1017-25.

57. Morrison, D.A., et al., Effects of aerobic training on exercise tolerance and echocardiographic dimensions in untrained postmenopausal women. Am Heart J, 1986. **112**(3): p. 561-7.

58. Neves, L.M., et al., Functional training reduces body fat and improves functional fitness and cholesterol levels in postmenopausal women: a randomized clinical trial. J Sports Med Phys Fitness, 2017. **57**(4): p. 448-456.

59. Nicholson, V.P., et al., Low-Load Very High-Repetition Resistance Training Attenuates Bone Loss at the Lumbar Spine in Active Post-menopausal Women. Calcif Tissue Int, 2015. **96**(6): p. 490-9.

60. Nunes, P.R., et al., Effect of resistance training on muscular strength and indicators of abdominal adiposity, metabolic risk, and inflammation in postmenopausal women: controlled and randomized clinical trial of efficacy of training volume. Age (Dordr), 2016. **38**(2): p. 40.

61. Oh, S.L., et al., Effects of rural community-based integrated exercise and health education programs on the mobility function of older adults with knee osteoarthritis. Aging Clin Exp Res, 2021. **33**(11): p. 3005-3014.

62. Oh, S.L., et al., Effects of an integrated health education and elastic band resistance training program on physical function and muscle strength in community-dwelling elderly women: Healthy Aging and Happy Aging II study. Geriatr Gerontol Int, 2017. **17**(5): p. 825-833.

63. Orsatti, F.L., et al., Plasma hormones, muscle mass and strength in resistance-trained postmenopausal women. Maturitas, 2008. **59**(4): p. 394-404.

64. Paolillo, F.R., et al., Phototherapy during treadmill training improves quadriceps performance in postmenopausal women. Climacteric, 2014. **17**(3): p. 285-93.

65. Park, J., Y. Kwon, and H. Park, Effects of 24-Week Aerobic and Resistance Training on Carotid Artery Intima-Media Thickness and Flow Velocity in Elderly Women with Sarcopenic Obesity. J Atheroscler Thromb, 2017. **24**(11): p. 1117-1124.

66. Park, S., et al., Influence of Isometric Exercise Combined With Electromyostimulation on Inflammatory Cytokine Levels, Muscle Strength, and Knee Joint Function in Elderly Women With Early Knee Osteoarthritis. Front Physiol, 2021. **12**: p. 688260.

67. Pospieszna, B., et al., Influence of 12-week Nordic Walking training on biomarkers of endothelial function in healthy postmenopausal women. J Sports Med Phys Fitness, 2017. **57**(9): p. 1178-1185.

68. Pu, C.T., et al., Randomized trial of progressive resistance training to counteract the myopathy of chronic heart failure. J Appl Physiol (1985), 2001. **90**(6): p. 2341-50.

69. Rashti, B.A., et al., The influence of concurrent training intensity on serum irisin and abdominal fat in postmenopausal women. Prz Menopauzalny, 2019. **18**(3): p. 166-173.

70. Raso, V., et al., Effect of resistance training on immunological parameters of healthy elderly women. Med Sci Sports Exerc, 2007. **39**(12): p. 2152-9.

71. Rezende, R.E., et al., Randomized clinical trial: benefits of aerobic physical activity for 24 weeks in postmenopausal women with nonalcoholic fatty liver disease. Menopause, 2016. **23**(8): p. 876-83.

72. Rhodes, E.C., et al., Effects of one year of resistance training on the relation between muscular strength and bone density in elderly women. Br J Sports Med, 2000. **34**(1): p. 18-22.

73. Ribeiro, A.S., et al., Resistance Training Improves a Cellular Health Parameter in Obese Older Women: A Randomized Controlled Trial. J Strength Cond Res, 2020. **34**(10): p. 2996-3002.

74. Ribeiro, A.S., et al., Resistance training prescription with different load-management methods improves phase angle in older women. Eur J Sport Sci, 2017. **17**(7): p. 913-921.

75. Saarto, T., et al., Effect of supervised and home exercise training on bone mineral density among breast cancer patients. A 12-month randomised controlled trial. Osteoporos Int, 2012. **23**(5): p. 1601-12.

76. Santos, W., et al., Once a Week Resistance Training Improves Muscular Strength in Breast Cancer Survivors: A Randomized Controlled Trial. Integr Cancer Ther, 2019. **18**: p. 1534735419879748.

77. Seo, M.W., et al., Effects of 16 Weeks of Resistance Training on Muscle Quality and Muscle Growth Factors in Older Adult Women with Sarcopenia: A Randomized Controlled Trial. Int J Environ Res Public Health, 2021. **18**(13).

78. Shaw, B.S., et al., Anthropometric and cardiovascular responses to hypertrophic resistance training in postmenopausal women. Menopause, 2016. **23**(11): p. 1176-1181.

79. Sipilä, S. and H. Suominen, Effects of strength and endurance training on thigh and leg muscle mass and composition in elderly women. J Appl Physiol (1985), 1995. **78**(1): p. 334-40.

80. Son, W.M., E.J. Pekas, and S.Y. Park, Twelve weeks of resistance band exercise training improves age-associated hormonal decline, blood pressure, and body composition in postmenopausal women with stage 1 hypertension: a randomized clinical trial. Menopause, 2020. **27**(2): p. 199-207.

81. Son, W.M., et al., Combined exercise reduces arterial stiffness, blood pressure, and blood markers for cardiovascular risk in postmenopausal women with hypertension. Menopause, 2017. **24**(3): p. 262-268.

82. Souza, M.F., et al., Effect of resistance training on phase angle in older women: A randomized controlled trial. Scand J Med Sci Sports, 2017. **27**(11): p. 1308-1316.

83. Strandberg, E., et al., Resistance Training Alone or Combined With N-3 PUFA-Rich Diet in Older Women: Effects on Muscle Fiber Hypertrophy. J Gerontol A Biol Sci Med Sci, 2019. **74**(4): p. 489-494.

84. Taaffe, D.R., et al., Comparative effects of high- and low-intensity resistance training on thigh muscle strength, fiber area, and tissue composition in elderly women. Clin Physiol, 1996. **16**(4): p. 381-92.

85. Taaffe, D.R., et al., The effect of hormone replacement therapy and/or exercise on skeletal muscle attenuation in postmenopausal women: a yearlong intervention. Clin Physiol Funct Imaging, 2005. **25**(5): p. 297-304.

86. Morteza Taheri, K.I., The Exercise-Induced Weight Loss Improves Self-Reported Quality of Sleep in Obese Elderly Women with Sleep Disorders. Sleep and Hypnosis, 2018.

87. Tan, S., et al., Exercise Training at Maximal Fat Oxidation Intensity for Older Women with Type 2 Diabetes. Int J Sports Med, 2018. **39**(5): p. 374-381.

88. Tomeleri, C.M., et al., Resistance training reduces metabolic syndrome and inflammatory markers in older women: A randomized controlled trial. J Diabetes, 2018. **10**(4): p. 328-337.

89. Tsourlou, T., et al., The effects of a twenty-four-week aquatic training program on muscular strength performance in healthy elderly women. J Strength Cond Res, 2006. **20**(4): p. 811-8.

90. Tsutsumi, T., et al., Comparison of high and moderate intensity of strength training on mood and anxiety in older adults. Percept Mot Skills, 1998. **87**(3 Pt 1): p. 1003-11.

91. Urzi, F., et al., Effects of Elastic Resistance Training on Functional Performance and Myokines in Older Women-A Randomized Controlled Trial. J Am Med Dir Assoc, 2019. **20**(7): p. 830-834.e2.

92. van Gemert, W.A., et al., Quality of Life after Diet or Exercise-Induced Weight Loss in Overweight to Obese Postmenopausal Women: The SHAPE-2 Randomised Controlled Trial. PLoS One, 2015. **10**(6): p. e0127520.

93. Vélez-Toral, M., et al., Improvements in Health-Related Quality of Life, Cardio-Metabolic Health, and Fitness in Postmenopausal Women After an Exercise Plus Health Promotion Intervention: A Randomized Controlled Trial. J Phys Act Health, 2017. **14**(5): p. 336-343.

94. Verschueren, S.M., et al., Effect of 6-month whole body vibration training on hip density, muscle strength, and postural control in postmenopausal women: a randomized controlled pilot study. J Bone Miner Res, 2004. **19**(3): p. 352-9.

95. von Stengel, S., et al., Effect of whole-body vibration on neuromuscular performance and body composition for females 65 years and older: a randomized-controlled trial. Scand J Med Sci Sports, 2012. **22**(1): p. 119-27.

96. Wen, H.J., et al., Effects of short-term step aerobics exercise on bone metabolism and functional fitness in postmenopausal women with low bone mass. Osteoporos Int, 2017. **28**(2): p. 539-547.

97. Son, W.M. and J.J. Park, Resistance Band Exercise Training Prevents the Progression of Metabolic Syndrome in Obese Postmenopausal Women. J Sports Sci Med, 2021. **20**(2): p. 291-299.

98. Wong, A., et al., The effects of stair climbing on arterial stiffness, blood pressure, and leg strength in postmenopausal women with stage 2 hypertension. Menopause, 2018. **25**(7): p. 731-737.

99. Wong, A., et al., The effects of swimming training on arterial function, muscular strength, and cardiorespiratory capacity in postmenopausal women with stage 2 hypertension. Menopause, 2018. **26**(6): p. 653-658.

100. Yoo, E.J., T.W. Jun, and S.A. Hawkins, The effects of a walking exercise program on fall-related fitness, bone metabolism, and fall-related psychological factors in elderly women. Res Sports Med, 2010. **18**(4): p. 236-50.

101. Yoon, D.H., et al., Effect of elastic band-based high-speed power training on cognitive function, physical performance and muscle strength in older women with mild cognitive impairment. Geriatr Gerontol Int, 2017. **17**(5): p. 765-772.
